# Lampshade web spider *Ectatosticta davidi* chromosome-level genome assembly provides evidence for its phylogenetic position

**DOI:** 10.1038/s42003-023-05129-x

**Published:** 2023-07-18

**Authors:** Zheng Fan, Lu-Yu Wang, Lin Xiao, Bing Tan, Bin Luo, Tian-Yu Ren, Ning Liu, Zhi-Sheng Zhang, Ming Bai

**Affiliations:** 1 Key Laboratory of Zoological Systematics and Evolution, Institute of Zoology, Chinese Academy of Sciences, 100101 Beijing, China; 2 School of Life Sciences, Southwest University, 400700 Chongqing, China; 3 Northeast Asia Biodiversity Research Center, Northeast Forestry University, 150040 Harbin, China; 4 University of Chinese Academy of Sciences, 100049 Beijing, China

**Keywords:** Genome, Molecular evolution

## Abstract

The spider of *Ectatosticta davidi*, belonging to the lamp-shade web spider family, Hypochilidae, which is closely related to Hypochilidae and Filistatidae and recovered as sister of the rest Araneomorphs spiders. Here we show the final assembled genome of *E. davidi* with 2.16 Gb in 15 chromosomes. Then we confirm the evolutionary position of Hypochilidae. Moreover, we find that the GMC gene family exhibit high conservation throughout the evolution of true spiders. We also find that the MaSp genes of *E. davidi* may represent an early stage of MaSp and MiSp genes in other true spiders, while CrSp shares a common origin with AgSp and PySp but differ from MaSp. Altogether, this study contributes to addressing the limited availability of genomic sequences from Hypochilidae spiders, and provides a valuable resource for investigating the genomic evolution of spiders.

## Abstract (web summary)

The assembly of the *E. davidi* genomic sequence suggests that *E. davidi* might represent the earliest diversified group among the true spiders and provides a valuable genomic resource for further biological and genetic studies on spiders.

## Introduction

Spiders (Araneae) are one of the most successful terrestrial arthropod groups, with high diversity (>51,000 described species) worldwide^1^. The vast majority of spiders (>93%) belong to the infraorder Araneomorphae (suborder Opisthothelae), also known as true or modern spiders. The lampshade web spider family Hypochilidae had ever been thought as the sister group of all other true spiders^2–5^. However, recent phylogenomic analysis confirmed that it was the sister group of the crevice weaver spider family Filistatidae and the sistership of (Hypochilidae + Filistatidae) with Haplogynae or Synspermiata^6–8^, a true spider clade with relatively simple genitalia.

Morphologically, several primitive characters of Araneomorphae have been presented in Hypochilidae (*Ectatosticta* and *Hypochilus*), such as two pairs of booklungs, a wide and short, undivided cribellum, and simple genitalia^3,9^. However, there is little molecular evidence representing this primitive spider group, which include the mitochondrial genome of *Hypochilus thorelli* and some conserved genes of both genera for phylogenetic analysis or species delimitation^8,10–16^.

Genomic data offers a large amount of genetic information for species, enabling a deeper understanding of their evolution, adaptation, and serving as a basis for further investigations into their biological mechanisms and practical applications. Currently, there are a total of 30 publicly accessible spider genome sequences by April, 2023 (Supplementary Table 1). These resources have made important contributions to research on adaptive evolution^17–24^, behavior^25^, and unique spider traits like silk production^26–29^ and venom composition^30–32^. However, it is important to note that the available spider genome data represent only a fraction of the genetic diversity found within the vast number of spider species, amounting to less than 1000th of the total species. This highlights the pressing need for further genomic studies to encompass a broader range of spiders and enhance our understanding of their genetic landscape.

The spider *Ectatosticta davidi* (Supplementary Fig. 1), belongs to the hypochilid genus, *Ectatosticta* from China, which can be usually found in valleys above 1000 m of altitude, building a large sheet web under/inside stones, caves, earth crevices, and tree cavities near rivers or in humid habitat^14^. The *Ectatosticta* spiders often hang themselves under their web, like spiders of Pimoidae and Psechridae. Here, we obtained a high-quality genome sequence of *E. davidi*, which is helpful to get more genetic characteristics, refine the phylogenetic position of this group, and further our understanding of their environmental adaptative evolution.

## Results

### Chromosome-level genome sequencing, assembly, and annotation

We obtained ~115 Gb of data via Illumina short-read sequencing, 193 Gb via PacBio long-read sequencing, and 278 Gb via Hi-C read sequencing, corresponding to 53×, 89×, and 128× genome coverage, respectively.

**Fig. 1:**
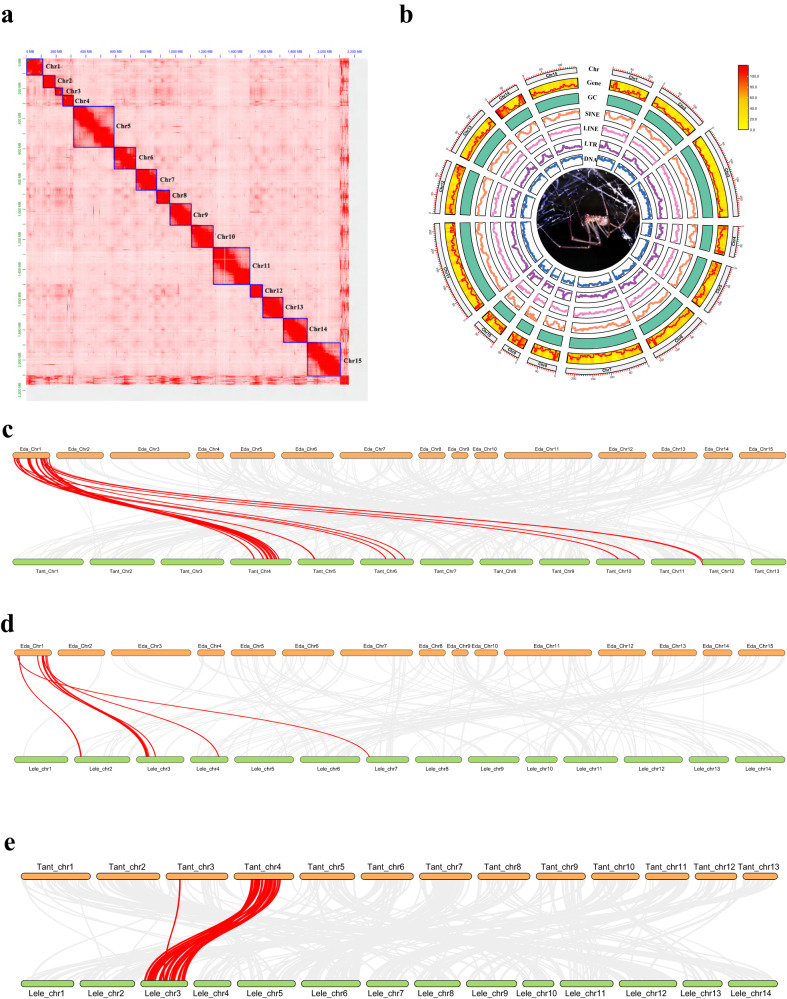
The genome fetures of *Ectatosticta davidi* . **a** The Hi-C assisted assembly of *E. davidi*. **b** Distribution of chromosomal elements of *E. davidi*. The inner ring contains a picture of *E. davidi*. The outer rings of the circle represent means bellow, respectively: Chr chromosomes, Gene distribution of genes, GC GC content, SINE short interspersed nuclear element, LINE long interspersed nuclear elements, LTR long terminal repeat, DNA DNA transposable elements. **c**–**e** Synteny analysis between *E. davidi*, *T. antipodiana*, and *L. elegans*. Red lines between the two species was as the sample of the interchromosomal rearrangements.

Evaluation of genome characteristics indicated that the genome was ~1.9 Gb, and the heterozygosity was 1.19–1.29% (Supplementary Fig. 2), thus suggesting a complex genome of *E. davidi*. We obtained a draft genome assembly of 2.16 Gb in length with a scaffold N50 value of 146.18 Mb (Supplementary Table 2), and the complete BUSCO analysis was 95.4% (of which 90.8% was single-copy), which ensured its suitability for downstream analysis. Each step of genome assembly is shown in Supplementary Table 2. The de novo genome assembly of *E. davidi* mainly comprised 15 chromosomes (Fig. 1a).

A total of 1.44 Gb of repeat sequences, accounting for 66.73% of the *E. davidi* genome, were identified (Supplementary Table 3 and Fig. 1b). Specifically, 2.54% of repeat sequences were short interspersed nuclear elements (SINEs), 10.69% long interspersed nuclear elements (LINEs), 2.16% long terminal repeats (LTRs), 10.83% DNA transposons, and 36.21% unclassified. In addition, we identified 11.66 Mb of small RNA sequences, 3.73 Mb of satellites, 21.50 Mb of simple repeats, and 1.79 Mb of low complexity.

Three methods were used for gene prediction, and 15,651 genes were annotated. A total of 15,392 genes (98.34%) were anchored to 15 chromosomes. The average gene length was 33,888.9 bp, and the average intron length was 3,735.67 bp. At the protein level, BUSCO completeness score was 90.4% (*n* = 1013), including 832 (82.1%) single-copy genes and 84 (8.3%) duplicated genes. Approximately 15,600 (~99.67%) genes were functionally annotated using the SwissProt or TrEMBL databases. InterProScan and EggnOG analyses identified protein domains for 13,670 (87.34%) genes, 11,296 GO terms, 10,259 KEGG ko terms, 6357 KEGG pathways, 14,090 COG categories, and 3457 enzyme codes. We also identified 10,866 noncoding RNAs, including 270 miRNAs, 81 rRNAs, 305 snRNAs, 6 ribozymes, and 753 other RNAs. A total of 9451 tRNAs were identified, which accounted for the majority of noncoding RNAs.

We noticed that the *piggyBac* transposases are greatly expanded in the *E. davidi* genome (Supplementary Tables 3 and 5). We identified 58 *piggyBac* genes in the *E. davidi* genome, including seven PGBD1, three PGBD2, 19 PGBD3, and 29 PGBD4 genes (Supplementary Table 6). And the *piggyBac* genes were distributed among all chromosomes in the *E. davidi* genome (Supplementary Fig. 3).

### Synteny analysis among *E. davidi*, *Trichonephila antipodiana* (Araneidae), and *Latrodectus elegans* (Theridiidae)

Synteny analysis for *E. davidi* and *T. antipodiana* showed that the genomes of the two species had 292 syntenic blocks with 4547 collinear genes (Fig. 1c), that for *E. davidi* and *L. elegans* showed 140 syntenic blocks with 2056 collinear genes (Fig. 1d), and that for *L. elegans* and *T. antipodiana* showed 327 syntenic blocks with 5857 collinear genes (Fig. 1e).

### Phylogenetic analysis

**Fig. 2:**
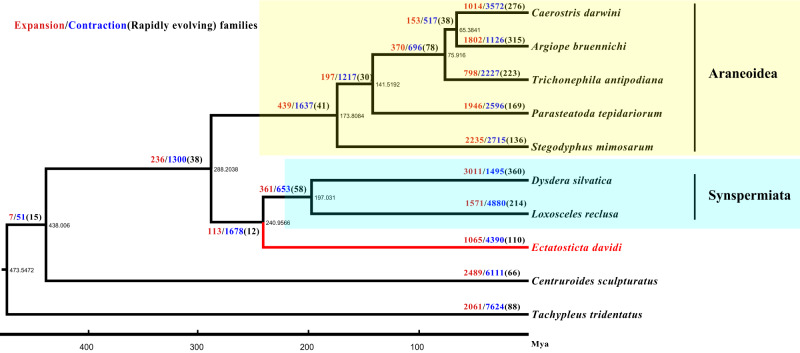
Phylogenetic and expansion gene family analyses of *E. davidi*. Phylogenetic relationship between *E. davidi* and other representative Arachnida species. The divergence times among different species are shown at the bottom. Node values indicate gene families showing expansion (blue), contraction (red), and rapid evolution (black in brackets).

A total of 347 single-copy genes were used to construct phylogenetic relationships (Fig. 2). The phylogenetic tree revealed that the divergence time of true spiders is 288.20 Ma, whereas the lampshade web spider emerged in 240.96 Ma.

A total of 1065 expanded gene families and 4390 contracted gene families were identified in *E. davidi*. Among them, 110 gene families underwent rapid evolution (*P* < 0.05), with 55 rapidly evolving expanding families and 55 rapidly evolving contracting families (Fig. 2).

### GMC gene family

The *Gld* genes, which belong to the enzymes of the glucose-methanol-choline (GMC) oxidoreductase family, were greatly expanded in the *E. davidi* genome, compared with other seven representative spider species. This is the first report of GMC gene family in spiders. In the *E. davidi* genome, the *GMC* gene showed an expansion of 44 copies. We also identified *GMC* genes in other spiders, including 27 in the genome of *Argiope bruennichi* (Araneidae), 19 in *Caerostris darwini* (Araneidae), 30 in *Caerostris extrusa* (Araneidae), 34 in *Nephila pilipes* (Araneidae), 37 in *Parasteatoda tepidariorum* (Theridiidae), 14 in *Stegodyphus dumicola* (Eresidae), 13 in *Stegodyphus mimosarum* (Eresidae), 16 in *Trichonephila antipodiana* (Araneidae), and 25 in *Trichonephila clavipes* (Supplementary Fig. 4b and Supplementary Table 7).

**Fig. 3:**
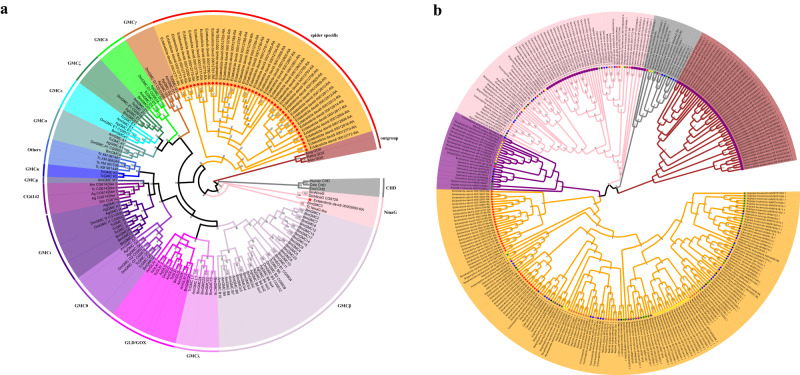
GMC gene family analysis in *E. davidi*. **a** Phylogenetic tree of GMC gene family in *E. davidi* and other representative Arachnida species. Ag (*A. gambiae*), Am (*A. mellifera*), Anig (*A. niger*), Aory (*A. oryzae*), Cele (*C. elegans*), Dm (*D. melanogaster*), Ecol (*E. coli*), Pama (*P. amagasakiense*), Tc (*T. castaneum*). Red star represents *E. davidi*. **b** Phylogenetic tree of GMC gene family in *E. davidi* and other representative spider species. The species include *A. bruennichi*, *C. darwini*, *C. extrusa*, *N. pilipes*, *P. tepidariorum*, *S. dumicolals*, *S. mimosarum*, *T. antipodiana*, and *T. clavipes*. The outgroup species is Scorpiones *C. sculpturatus*.

We build a phylogenetic tree of the *GMC* genes between the *E. davidi* and some representative insects such as fruit fly *D. melanogaster*, mosquito *Anopheles gambiae*, the honeybee *A. mellifera*, and the flour beetle *Tribolium castaneum* (Fig. 3a). The *GMC* genes of *E. davidi* were separated into two subfamilies: *NinaG*, which is also found in insects, and an unknown spider-specific subfamily.

To analyze the spider-specific *GMC* genes, we build an ML tree with eight spiders and the Arizona bark scorpion *C. sculpturatus* as the outgroup (Fig. 3b). The tree showed four major clades (excluding outgroup sequences), and the sequences clustered in each clade were classified as subfamilies. Bootstrap resampling analysis indicated that the clustering of these subfamilies was reliable. We found that the *GMC* genes of *E. davidi* in most subfamilies were at the position of the sister to the rest genes, which is the same with its phylogenetic position. In this study, we did not name these spider-specific subfamilies.

To investigate the function of *GMC* genes in spiders, we examined the expression of these genes. Because of insufficient tissue from *E. davidi* for RNA sequencing, we downloaded the *P. tepidariorum* transcriptome at different stages (stages 1–10) (Supplementary Fig. 4c). In *P. tepidariorum*, some *GMC* genes, such as LOC107453087, were expressed at all stages (Supplementary Fig. 4c). Some genes were expressed during the early stages (stages 1 and 2), such as LOC107443921 and LOC107453228, and some genes were expressed in late stages (stages 6, 7, 8, and 10), such as LOC107438235 and LOC107449348 (Supplementary Fig. 4c). In addition, the distribution of *GMC* genes in the *E. davidi* genome was on chr1, chr4, and chr6 (Supplementary Fig. 4a).

### Ir/iGluR and cytochrome P450 gene family

**Fig. 4:**
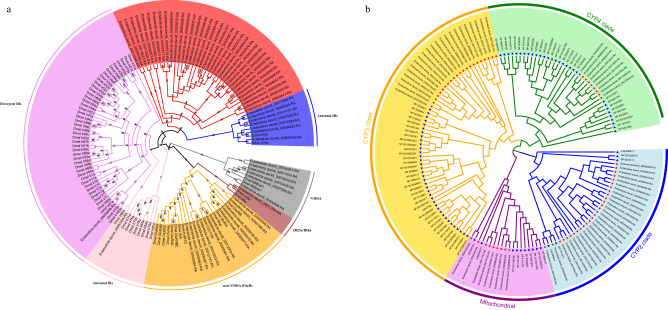
Phylogenetic analysis of IR/iGluR and P450 gene families in *E. davidi*. **a** Phylogenetic tree of IR/iGluR gene family in *E. davidi* and *D. melanogaster*. **b** Phylogenetic tree of P450 gene family in *E. davidi* and *D. melanogaster*.

We identified 101 *IR*/*iGluR* genes in the *E. davidi* genome, which include 82 complete genes: 59 exhibiting the specific domain signature of the ionotropic glutamate receptors (IPR001320) and 8 with all three characteristic domains (ATD domain, PF01094; LBD-domain, PF10613; and LCD-domain, PF00060). We used the complete *IR*/*iGluR* genes in *E. davidi* to perform a phylogenetic analysis, with *D. melanogaster* as the outgroup. The phylogenetic tree showed that the *IR*/*iGluR* genes belonged to some gene groups, including NMDA, non-NMDA iGluR, Divergent IR, Antennal IR, IR25a/IR8a, and one special *E. davidi* expansion group, which was a sister group to the *Antennal IR* group (Fig. 4a). In the *E. davidi* genome, the *IR*/*iGluR* genes were distributed among all chromosomes, except chr10 (Supplementary Fig. 5).

We identified 68 *P450* genes comprising four major classes: the CYP2 clade (28 genes), mitochondrial P450 clade (9), CYP3 clade (22), and CYP4 clade (9). We reconstructed an ML tree with *P450* genes from *E. davidi*, with *D. melanogaster* as the outgroup (Fig. 4b). The CYP2 and CYP3 clade genes showed expansion when compared to *D. melanogaster*.

### Silk and venom genes in *E. davidi*

Silk is an important tool for spider to forage, locomote, nest, mate, egg protect, and communication^33^. The venom is utilized by spiders in defensive and predatory interactions^34^. We identified the silk and toxin genes in *E. davidi*.

**Fig. 5:**
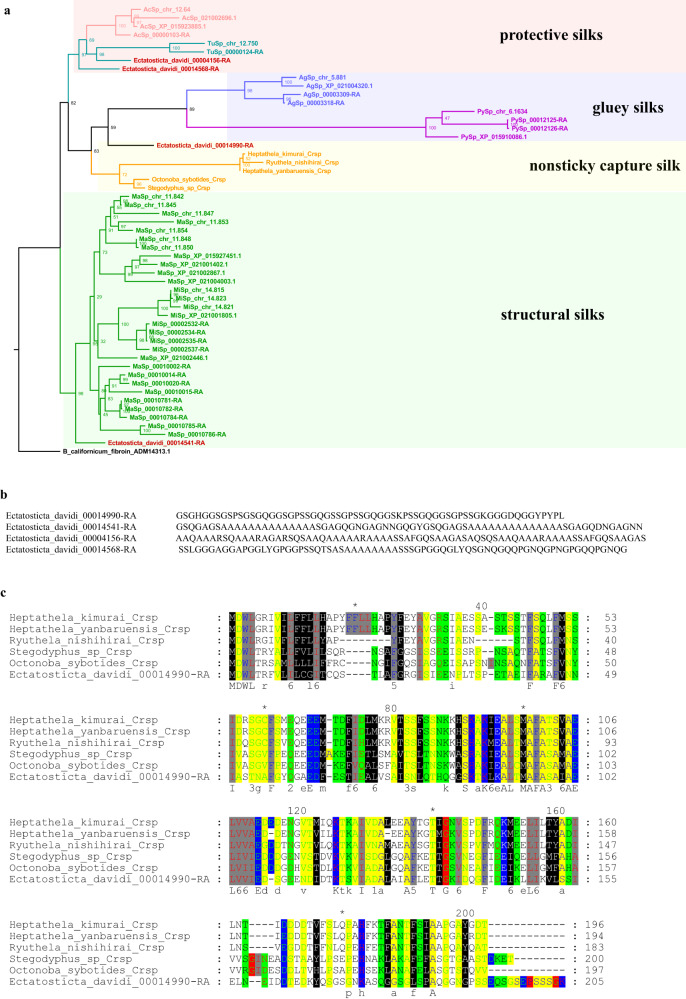
Spider silk gene analysis in *E. davidi*. **a** Phylogenetic analysis of spider silk gene in *E. davidi*. The N-terminal domains of silk genes in the species (such as *E. davidi, T. antipodiana, P. tepidariorum*, and *L. elegans*) were used for Maximum-likelihood (ML) phylogenetic tree. **b** Repeat regions of spider silk genes in *E. davidi*. **c** The spidroin N-terminal domains of the *E. davidi* gene Ectatosticta_davidi_00014990 bear close resemblance to CrSp sequence of some Mesothelae species including Liphistiidae (*H. kimurai*, *H. yanbaruensis*, *R. nishihirai*), Eresidae (*Stegodyphus sp*.), and Uloboridae (*O. sybotides*).

In *E. davidi*, four silk genes were identified: TuSp, MaSp, AcSp, and CrSp (Supplementary Table 8). Phylogenetic analysis of the N-terminal sequence revealed that Ectatosticta_davidi_00014541 was at sister group of MaSp clade, and the gene Ectatosticta_davidi_00004156 was at sister group of the TuSp clade (Fig. 5a). The repeat regions of the four silk genes are shown in Fig. 5b. We also compared the N-terminal domain of the CrSp gene of *E. davidi* with the “primitive” spider species *Heptathela kimurai* (Liphistiidae), *Heptathela yanbaruensis* (Liphistiidae), *Ryuthela nishihirai* (Liphistiidae), and the diverse RTA clade *Stegodyphus sp*. (Eresidae) and *Octonoba sybotides* (Uloboridae). We found that these sequences bear a close similarity (Fig. 5c). The amino acid composition of the spider silk protein gene was also identified, and the top three amino acids were Gly, Ser, and Ala (Supplementary Fig. 6).

**Fig. 6:**
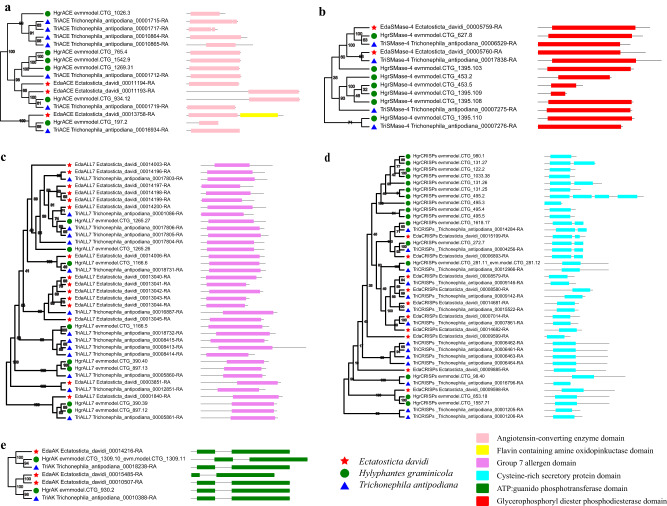
Phylogenetic analysis and protein domain structure of toxin gene families in *E. davidi*, *H. graminicola*, and *T. antipodiana*. **a** Phylogenetic analysis and protein domain structure of ACE toxin gene family. **b** Phylogenetic analysis and protein domain structure of AK toxin gene family. **c** Phylogenetic analysis and protein domain structure of ALL7 toxin gene family. **d** Phylogenetic analysis and protein domain structure of SMase-4 toxin gene family. **e** Phylogenetic analysis and protein domain structure of CRISPs toxin gene family. Red star represent the toxin genes of *E. davidi*. Green circle represent the toxin genes of *H. graminicola*. Blue triangle represent the toxin genes of *T. antipodiana*. The pink, yellow, purple, sky blue, green and red rectangle respectively represent the Angiotensin-converting enzyme domain, Flavin containing amine oxidopinkuctase domain, Group 7 allergen domain, Cysteine-rich secretory protein domain, ATP:guanido phosphotransferase domain, Glycerophosphoryl diester phosphodiesterase domain.

In total, 45 toxin genes were identified in the *E. davidi* genome (Supplementary Table 9) and classified in seven types: angiotensin-converting enzyme (ACE), sphingomyelin phosphodiesterase D (Smase-4), group 7 allergen (ALL7), cysteine-rich secretory proteins (CRISPs), and arginine kinase (AK). The phylogenetic analyses of ACE, AK, ALL7, SMase-4, and CRISPs toxin gene families and the protein domain structures of *E. davidi*, *H. graminicola*, and *T. antipodiana* are shown in Fig. 6. Phylogenetic analysis showed that the toxin genes in *E. davidi* were correctly identified (Fig. 6). The toxin genes in the *E. davidi* genome were distributed on all chromosomes (Supplementary Fig. 7).

## Discussion

### The high-quality genome sequence of *E. davidi* provides a valuable resource for studying spiders’ evolution and adaptability

To date, the majority of whole genome-sequenced spiders come from well-studied spider groups such as Araneoidea (Araneidae, Tetragnathidae, Theriidae, Linyphiidae)^19,23,25,28,29,31,32,35–41^ and the marronoid clade (Lycosidae, Pisauridae)^42^. A few genomes have been obtained from Synspermiata (Drymusidae, Dysderidae)^18^ and Mygalomorphae (Theraphosidae)^43^ (Supplementary Table 1). Notably, the genome sequence of *E. davidi* represents the first high-quality genome from the Hypochilidae family. It provides crucial genetic data to advance our understanding of spider evolution, adaptability, and biology. The genome of *E. davidi* measures 2.16 Gb in size, with a BUSCO quality evaluation of 95.4%. Furthermore, it was assembled into 15 chromosomes. These findings demonstrate that this genome is of moderate size, exhibits high-quality sequencing, and possesses a moderate number of chromosomes compared with other spiders (Supplementary Table 1).

### The genome of *E. davidi* supports the previous phylogenomics hypothesis

Phylogenetic analysis was performed to determine the phylogenetic position of *E. davidi* (Hypochiidae) based on available genome data of spiders, including two species of Synspermiata (*Dysdera silvatica* and *Loxosceles reclusa*) and five species of Entelegynae (*C. darwini*, *A. bruennichi*, *Trichonephila antipodiana*, *Parasteatoda tepidariorum*, and *Stegodyphus mimosarum*). Theoretically, genomic data of representatives of the suborder Mesothelae, the infraorder Mygalomorphae, and the family Filistatidae should be included. However, the genomes of Mesothelae and Filistatidae are presently unavailable, and the genome contiguity quality of the Mygalomorphae (Theraphosidae, *A. geniculata*) was low with Contig N50 of 0.54 kbp (Supplementary Table 1). The result (Fig. 2a) showed that the lampshade web spider is a sister group of Synspermiata, in accordance with several phylogenetic or phylogenomic results recently^6–8^. The phylogenomic results showed that the divergence time of Araneomorphae from their common ancestor might be Early Permian (288.20 Ma) while the lampshade web spider should be Early Triassic (240.96 Ma).

The evolutionary trajectory of diverging populations and likelihood of speciation can be heavily influenced by recombination^44^. Genomic rearrangements in animals have been broadly studied, and it has been suggested that synteny blocks and their composition (number of genes and their maximum and average size) correspond to phylogenetic distribution^45^. Synteny analysis was performed for *E. davidi* with two representative true spiders (*T. antipodiana* and *L. elegans*) (Fig. 1c–e). Compared to the number of collinear genes between *E. davidi* and the two spiders (*T. antipodiana* and *L. elegans*), there were more collinear genes between *E. davidi* and *T. antipodiana* than *L. elegans*. It seems most genes of *E. davidi* was “inherited” by other true spiders, although *T. antipodiana* (and maybe other true spiders) undergoes a long history and variety of interchromosomal rearrangements. Using the *E. davidi* chr1 as an example, most of the synteny blocks of *E. davidi* chr1 matched *T. antipodiana* chr4 (Fig. 1c) and L. *elegans* chr3 (Fig. 1d). *T. antipodiana* chr4 had a good genome synteny relationship with *L. elegans* chr3 (Fig. 1e). However, the number of synteny blocks between *E. davidi* chr1 and *T. antipodiana* chr4 was greater than *L. elegans* chr3, which may be related to the divergence time of these two species and their adaptation to the environment.

The phylogenetic tree of the GMC gene family among spiders showed that most of the related genes of *E. davidi* were located in the basal lineage of the phylogenetic tree of the four GMC subfamilies among spiders, indicating their highly conserved characteristics (Fig. 3b). In insects, four core genes (MCδ, ε, ζ, and θ) in the middle of the GMC cluster have remained in tandem and in the same orientation for hundreds of millions of years, strongly suggesting that this cluster is conserved^46^. Although the types of core genes among spiders and insects were different, *GMC* genes were partially or entirely conserved.

As spiders evolved, the types of silk refined and increased^47^. Mygalomorphae spiders are known to retain a higher number of ancestral states and are more primitive than the Araneomorphae. Spiders from this clade possess a simpler undifferentiated spinning apparatus consisting of uniform spigots that lead to 1–3 types of globular silk glands^48^. The most architecturally complex spider webs have evolved within a group of Araneoidea. For example, spiders of Araneidae have up to six morphologically distinct spinning glands^49^. If we consider the ecological functions of these silk proteins, the evolutionary relationships between these spiders can be determined. MaSps and MiSps are structural silks, AgSps and PySps form gluey silks, and AcSps and TuSps are both used to produce protective sacs for prey and eggs. Previous studies showed the presence of spidroin paralogs prior to the divergence of Mygalomorph and Araneomorph spiders, for Mygalomorph Spidroin 2 from *Ancylometes juruensis* (Ctenidae) clustered together within orbicularian MaSp2 sequences^50–53^. From the phylogenetic tree of spidroin genes (Fig. 5a), we found that TuSp, AcSp, MaSp, and CrSp of *E. davidi* were all located in the basal lineage of each clade. If we consider *E. davidi* as primitive, MaSp and MiSp may have the same origin from similar MaSp genes of *E. davidi* (Ectatosticta_davidi_00014541-RA), AgSp and PySp from similar CrSp genes of *E. davidi* (Ectatosticta_davidi_00014990-RA), and TuSp and AcSp from similar AcSp genes of *E. davidi* (Ectatosticta_davidi_00014568-RA). In addition, MaSp+Misp has a different origin from that of AcSp+TuSp+AgSp+PySp+Crsp. Our study supports the previously validated hypothesis.

### Gene family analysis suggests the unique adaptation evolution of *E. davidi*

The *piggyBac* transposable element is currently the vector of choice for transgenesis, enhancer trapping, gene discovery, and determination of gene function in both insects and mammals^54–56^. Genome sequence analysis of various species, such as silkworms (*Bombyx mori*), ants (*Camponotus floridanus* and *Harpegnathos saltator*), moths (*Macdunnoughia crassisigna*), and bats (*Myotis lucifugus*) shows that a number of previously unrecognized genes were derived from *piggyBac* transposases and other transposable elements^57–62^. The *piggyBac* transposases showed great expansion in the *E. davidi* genome (Supplementary Tables 3, 5, and 6), and is distributed on every chromosome (Supplementary Fig. 3). The expansion of *piggyBac* gene family in the *E. davidi* genome suggests that it may be helpful in creating new genes to adapt to the environment.

Compared to other spiders, there were more *GMC* genes in the *E. davidi* genome (Supplementary Table 7). The *GMC* genes of insects may have different roles in basic physiological processes and diverse metabolic processes, such as glucose metabolism, immunity, suppression of host plant defense responses, and basic physiological processes^46,63–65^. In spiders, there is little information on *GMC* genes. Phylogenetic analyses of spiders show that only the *NinaG* gene subfamily was similar to that of insects, whereas other genes belonged to the spider-specific GMC gene subfamily. Therefore, we conjecture that the spider’s *NinaG* gene may have the same function as that of an insect in the biogenesis of the rhodopsin chromophore, (3 S)-3-hydroxyretinal^66,67^. Analysis of different stages of *P. tepidariorum* transcriptome suggested that the spider *GMC* genes may be related to development (Supplementary Fig. 4c). The *GMC* genes were arranged in clusters in the *E. davidi* genome (Supplementary Fig. 4a), similar to that observed in insects^46^.

Chemoreception is important for animals to experience changes in nature. The iGluR superfamily is a large and ancient gene family, and the IR family is a variant lineage of the iGluR superfamily of ligand-gated ions^68^. The functional roles of *IR*/*iGluRs* are related to the sensing of hearing, olfaction, taste, temperature, and humidity^69–73^. Phylogenetic analysis of *E. davidi* confirmed that some genes may play the same role as in insects. For example, Ectatosticta_davidi_00009759 was homologous to *IR76b* (Fig. 4a), which was reported to be broadly expressed in both olfactory gustatory neurons with diverse chemical specificities in insects^74^. The Ectatosticta_davidi_00009363 gene was homologous to *IR93a* (Fig. 4a), which has been reported to play an important role in both temperature and humidity sensing^74,75^. The *IR*/*iGluR* genes in *E. davidi* showed a special expansion clade, which was the sister clade with the Antennal IR clade (Fig. 4a). Evidence from *D. melanogaster* research has shown that changes in *IRs* may contribute to changes in preferred food and habitat^76^. Therefore, the special expansion clade may be related to spider adaptation to changes in food preferences and living habits. In *E. davidi*, 101 *IR*/*iGluR* genes were identified, whereas 435 are found in the spider *Dysdera sylvatica*^18^. We believe that the difference in *IR*/*iGluR* gene numbers between these two species may be related to their lifestyle. The spider *E. davidi* prefers living in stony debris in open, semi-open, and forest-covered habitats and obtains food through the web^77^, whereas *D. sylvatica* is an active nocturnal hunter of woodlice^78^.

For toxin genes, we identified 15 ALL7 genes in *E. davidi*, which were the most abundant in comparison to other species (Supplementary Table 10). ALL7 was first reported in the spider venom of *Hylyphantes graminicola*^32^. There are six ALL7 coding genes on chr4 and five genes on chr8 of *E. davidi* (Supplementary Fig. 7). These repeats may have been caused by gene duplication. Phylogenetic analysis of the toxin genes showed that those found in *E. davidi* were correctly identified (Fig. 6).

In conclusion, the assembly of the *E. davidi* genomic sequence is the first high-quality chromosome-level genome of Hypochilidae. Phylogenetic results based on genome and gene family (GMC and spidroin) of *E. davidi* and chromosomal synteny analyses confirm the position of Hypochilidae as recovered in the previous analysis. Our study supports the previously validated hypothesis that MaSp+Misp has a different origin from that of AcSp+TuSp+AgSp+PySp+Crsp. And the silk genes in *E. davidi* might be the most primitive spider silk genes of the true spiders. The expansion of gene families such as GMC (oxidoreductase enzymes, related to metabolism), *piggyBac* (one type of transposable element), Ir/iGluR (related to chemoreception), cytochrome P450 (related to metabolic detoxification) and spider venom ALL7 (related to prey) gene family, which is helpful for *E. davidi*’s to adaptation to the environment. In summary, this work provides a valuable genomic resource for further biological and genetic studies on spiders.

## Methods

### Sample collection and sequencing

Female specimens of *E. davidi* were collected from the Qinling Mountains, Chang’an District of Xi’an City, Shaanxi Province of China, in October 2020. Live samples were sent to Berry Genomics (Beijing, China) for sequencing. Briefly, spiders were cleaned and ground in liquid nitrogen. Genomic DNA was extracted using the Qiagen Blood & Cell Culture DNA Mini Kit, according to the protocol for PacBio and Illumina sequencing. PacBio sequencing was performed using PacBio Sequel II libraries with insert sizes of 20 kb, using a SMRTbell™ Template Prep Kit 1.0-SPv3. Paired-end reads (150 bp) were generated using the Illumina NovaSeq platform for survey analysis and Hi-C genome sequencing. Total RNA was extracted from a female adult spider of *E. davidi* using TRIzol (Invitrogen, U.S America) according to the manufacturer’s instructions and then sequenced on the Illumina NovaSeq platform.

### Quality control and genome survey analysis

Quality control of the raw Illumina data was performed using the BBTools suite v38.67^79^. The “clumpify.sh” tool was used to remove duplicates. The “bbduk.sh” tool was used to trim the reads’ ends to Q20 with reads shorter than 15 bp or with >5 Ns, poly-A/G/C tails of at least 10 bp, and overlapping paired reads.

To estimate the genome size and other characteristics, all filtered reads were used for the survey analysis. The k-mer distribution was estimated using “khist.sh”, and the 17-mer, 19-mer and 21-mer were all selected to investigate the genome size. The genome size was calculated using GenomeScope v1.0.0^80^, and the maximum k-mer coverage cutoff was set to 10,000. And we selected the results of 19-mer for its models fits best (Supplementary Fig. 2).

### Genome assembly and annotation

To obtain the high-quality *E. davidi* genome sequence, PacBio long reads were assembled into contigs using raven v1.6.1^81^. The heterozygous regions were reduced using Purge Haplotigs v1.1.0, with a 50% cutoff for identifying contigs as haplotigs^82^. Single-base errors in the genome assembly were corrected using the filtered Illumina reads by NextPolish (v1.3.1) over two rounds^83^. Minimap2 v2.12 was used as the read aligner^84^. The Hi-C sequencing reads generated a chromosome-level assembly of the genome using 3d-DNA and Juicer v1.6.2^85^.

Potential contaminant sequences were inspected using HS-BLASTN and BLAST+ (blastn) v2.7.1 against the NCBI nucleotide (nt) and UniVec databases^86^.

Repetitive element annotation of the *E. davidi* genome sequence was performed using a combination of ab initio and homology-based searching. The an-initio database was constructed using RepeatModeler v2.0.2^87^. We combined the an-initio database and repeat library (Repbase) as the reference repeat database. Repetitive elements were finally identified using RepeatMasker v4.1.2^88^.

Protein-coding gene annotation was performed using Maker pipline v3.01.03 by integrating ab initio, transcriptome-based, and protein homology-based evidence^89^. Previously, RNA-seq data were mapped to the *E. davidi* assembled genome sequence using HISAT2 v2.2.1^90^, and then assembled into transcripts using Stringtie v2.1.6^91^. For ab initio gene prediction, we used Augustus v3.4.0^92^ and GeneMark-ES/ET/EP v4.68_lic^93^. To accurately model the sequence properties, both gene finders were initially trained using the BRAKER v2.1.6 pipeline^94^, which uses the mapped transcriptome sequence data. For protein homology-based evidence, we downloaded the protein sequences of *Araneus ventricosus* (GCA_013235015.1), *Argiope bruennichi* (GCA_015342795.1), *Trichonephila inaurata madagascariensis* (GCA_019973955.1), *Trichonephila clavipes* (GCA_002102615.1), *Parasteatoda tepidariorum* (GCA_000365465.3), *Stegodyphus mimosarum* (GCA_000611955.2), *Caerostris extrusa* (GCA_021605095.1), *Caerostris darwini* (GCA_021605075.1), *Oedothorax gibbosus* (GCA_019343175.1), *Nephila pilipes* (GCA_019974015.1), *Drosophila melanogaster* (GCA_000001215.4), *Ixodes scapularis* (GCA_002892825.2), *Strigamia maritima* (GCA_000239455.1)*, Daphnia pulex* (GCA_900092285.2) from NCBI, and *Trichonephila antipodiana* from GigaDB^19^. For the Maker pipeline, the transcripts were provided as input via the “est” option and protein homology-based evidence as input via the “protein” option. And then removed redundant isoforms, kept the longest isoforms, and checked the possible errors for “two mRNAs extracted for single redundant seq”, and deleted proteins of length smaller than 50.

The predicted genes were functionally annotated using the following three ways: EggNOG-mapper v2.1.5^95^ was used to identify GO, EC (expression coherence), KEGG (Kyoto Encyclopedia of Genes and Genomes) pathways, KEGG orthologous groups (KOs), and COG (clusters of orthologous groups) with eggNOG v5.0 database. Diamond v2.0 was used to annotate homology-based gene functions with the SWISS-PROT and TrEMBL databases^96,97^. InterProScan v5.48-83.0 was used to screen protein sequences using the Pfam, Panther, Gene3D, Superfamily, and CDD databases^98–103^.

Noncoding RNA annotation was performed using infernal v1.1.4, and tRNAscan-SE v2.0.9^104,105^.

To assess the completeness of the genome or protein sequences of *E. davidi*, we used the BUSCO v5.2.2 pipeline^106^ and the arthropod reference set of arthropoda_odb 10 (*n* = 1013).

### Phylogenetic analyses and divergence time estimation

**S**ingle-copy orthologous gene families were identified by gene orthology analysis and then used for comparative genome analysis. For gene orthology analysis, we compared the protein-coding genes of *E. davidi* and other seven representative spider species, including Araneidae (*A. bruennichi, C. darwini*, and *T. antipodiana*), Theridiidae (*P. tepidariorum*), Eresidae (*S. mimosarum*), Sicariidae (*Loxosceles reclusa*), and Dysderidae (*Dysdera silvatica*), with Scorpiones (*C. sculpturatus*), and Xiphosura (*T. tridentatus*) as outgroups. Orthologous gene clusters were classified using OrthoFinder v2.5.4^107^.

Phylogenetic analysis was performed using previously identified single-copy genes. First, the protein sequences of single-copy genes were separately aligned using MAFFT v7.487, based on the L-INS-I strategy^108^. The resulting alignments were then fed to trimAl v1.4, to remove sites of unclear homology, using the heuristic method “automated1”^109^. All the well-trimmed single-copy genes in each species were concatenated to one super gene for each species using FASconCAT-G v1.04^110^. Finally, maximum-likelihood-based phylogenetic analysis was performed using IQ-TREE v2.1.3, with extended model selection followed by tree inference, model set by LG, with the number of partition pairs for the cluster algorithm, replicates for ultrafast bootstrap, and Shimodaira-Hasegawa (SH) approximate likelihood ratio tests of 1000, 10, and 1000, respectively^111^.

Fossil records were downloaded from the paleobiodb database (https://paleobiodb.org/) and TimeTree database (http://www.timetree.org/), with Nephilinae stem (43–47.8 Mya), Palpimanoidea stem (173.1–183.4 Mya), and split between scorpions and spiders (435–439 Mya). The divergence time was estimated using the MCMC Tree program in the PAML package v4.9j^112^ with the following parameters: independent clock rates; BD paras-related birth, death, and sampling rates of 1, 1, and 0.1, respectively; and Burnin, sampfreq, and nsample of 2000, 5, and 10000, respectively.

### Gene family evolution analysis

Café v4.2.1 and v5.0.0 were used to identify the likelihood of gene family expansion and contraction^113,114^. CAFE5 was used to predict the birth-death parameter lambda. The results were fed to CAFE4 and run with a P-value threshold of 0.01. And the conditional *P* value for each gene family was calculated. If the *P* values <0.05, the gene family was treated as having a significantly accelerated rate of expansion or contraction. And Gene families with >200 copies in one of the species were removed.

### Annotation of gene families

To manually annotate the genes of glucose-methanol-choline (GMC), *piggyBac*, ionotropic receptors and ionotropic glutamate receptors (Ir/iGluR) and P450 gene families, we initially downloaded the amino acid sequences of related species from the GenBank database, or related articles were used as the reference query. The reference GMC homologous protein sequences for *Drosophila melanogaster*, *Anopheles gambiae*, *Apis mellifera*, *Tribolium castaneum*, *Escherichia coli*, *Caenorhabditis elegans*, *Aspergillus niger*, *Aspergillus oryzae*, and *Penicillium amagasakiense* were downloaded from a previous study^46^. The reference *piggyBac* sequence accession number is shown in Supplementary Table 4. The reference for chemosensory sequence accession was downloaded from the dataset by Vizueta^115^.

We used the BITACORA pipeline to identify *Ir*/*iGluR* genes^116^. The “incomplete” (or “partial”) genes were checked for the length of the encoded protein, which contained less than 80% of the protein domain length characteristic of the family.

To identify *GMC*, *piggyBac*, and *P450* genes, we performed gene family analysis in three ways. First, a blastp-like search was performed by MMseqs2 v11 with four rounds of iteration^117^. Interproscan v5.48-83.0 was used to confirm specific conserved domains using the Pfam database^98^. Candidate proteins were filtered using MMseqs2 with a TBLATN-like search to delete invalid matches. And the method for identification P450 gene families was same with Fan^19^.

For the spidroin gene set, we downloaded protein sequences of the seven spidroin gene classes from the dataset by Arakawa^26^, and *Latrodectus elegans* data were downloaded from the dataset by Wang^31^. The reference CrSp gene was downloaded from the dataset by Arakawa^26^.

The reference toxin gene set was downloaded from the dataset by Zhu^32^.

### Phylogenetic analyses of the gene families

Multiple alignments of protein sequences were generated using MAFFT v7.487^108^, with the default parameters and necessary manual adjustments. The tree was constructed using IQ-TREE v2.1.3^111^. The tree was viewed and edited using FigTree v1.4.3 and the Evolview v3 webserver^118^. The position of the genes on the chromosome is shown using the online tool MG2C^119^.

### Synteny analysis

To look for changes in chromosomes among the ancient Araneomorphae spider and other true spiders, the synteny analysis between *E. davidi* and other spiders (including *T. antipodiana* and *L. elegans*) was carried out by MCScanX^120^, and the results are shown in TBtools^121^.

### GMC gene expression analysis

The RNA sequencing data^122^ of *P. tepidariorum* at different stages (stages 1–10) was downloaded from NCBI with the accession number of GSE112712 by SRA Toolkit v3.0.1 (http://www.ncbi.nlm.nih.gov/books/NBK158900/). The clean data was mapped to the reference genome by the software of HISAT2 v2.2.1^90^. The featureCounts v1.6.4 software was used to calculate the fragments per kilobase million (FPKM) values^123^. The R packages of DESeq2 were used to analyze the gene expression differences.

### Statistics and reproducibility

The genome assembly reported here was derived from the female of *E. davidi*. Our annotation pipeline was performed by integrating three evidence, such as ab initio, transcriptome-based, and protein homology-based evidence.

### Reporting summary

Further information on research design is available in the Nature Portfolio Reporting Summary linked to this article.

## Supplementary information

Supplementary information

Reporting Summary

## Data Availability

The sequencing data sets supporting the results of this article are available in NCBI (BioProjectID PRJNA853523). Illumina paired-end reads have been uploaded with SRA accession SRR19913594, Pacific Biosciences long-read data are associated with SRA accession SRR20336950, Hi-C data are available at SRR19905029, and RNA sequencing data generated for annotation are available with SRA accession SRR19913735. The genome assembly of *E. davidi* were deposited in ScienceDB Digital Repository with 10.57760/sciencedb.06872^124^. All other relevant data are available upon request.
