# Supplementary material for: Lampshade web spider Ectatosticta davidi chromosome-level genome assembly provides evidence for its phylogenetic position
Source: Commun Biol. 2023 Jul 18;6:748. doi: 10.1038/s42003-023-05129-x (PMC10354039; doi:10.1038/s42003-023-05129-x)
Supplement: Supplementary file 1 — Supplementary information [file 42003_2023_5129_MOESM1_ESM.pdf]

Lampshade web spider *Ectatosticta davidi* chromosome-level genome assembly provides evidence for its phylogenetic position

Zheng Fan[1, 2*], Lu-Yu Wang[2*], Lin Xiao[2*], Bing Tan[2], Bin Luo[2], Tian-Yu Ren[2], Ning Liu[1†], Zhi-Sheng Zhang[2†] and Ming Bai[1†]

1. Key Laboratory of Zoological Systematics and Evolution, Institute of Zoology, Chinese Academy of Sciences, Beijing 100101, China.

2. School of Life Sciences, Southwest University, Chongqing 400700, China.

*These authors contributed equally to this work.

†These authors jointly directed this work. Correspondence should be addressed to N.L. (liun@ioz.ac.cn); Z.Z. (zhangzs327@qq.com); M.B. (baim@ioz.ac.cn)

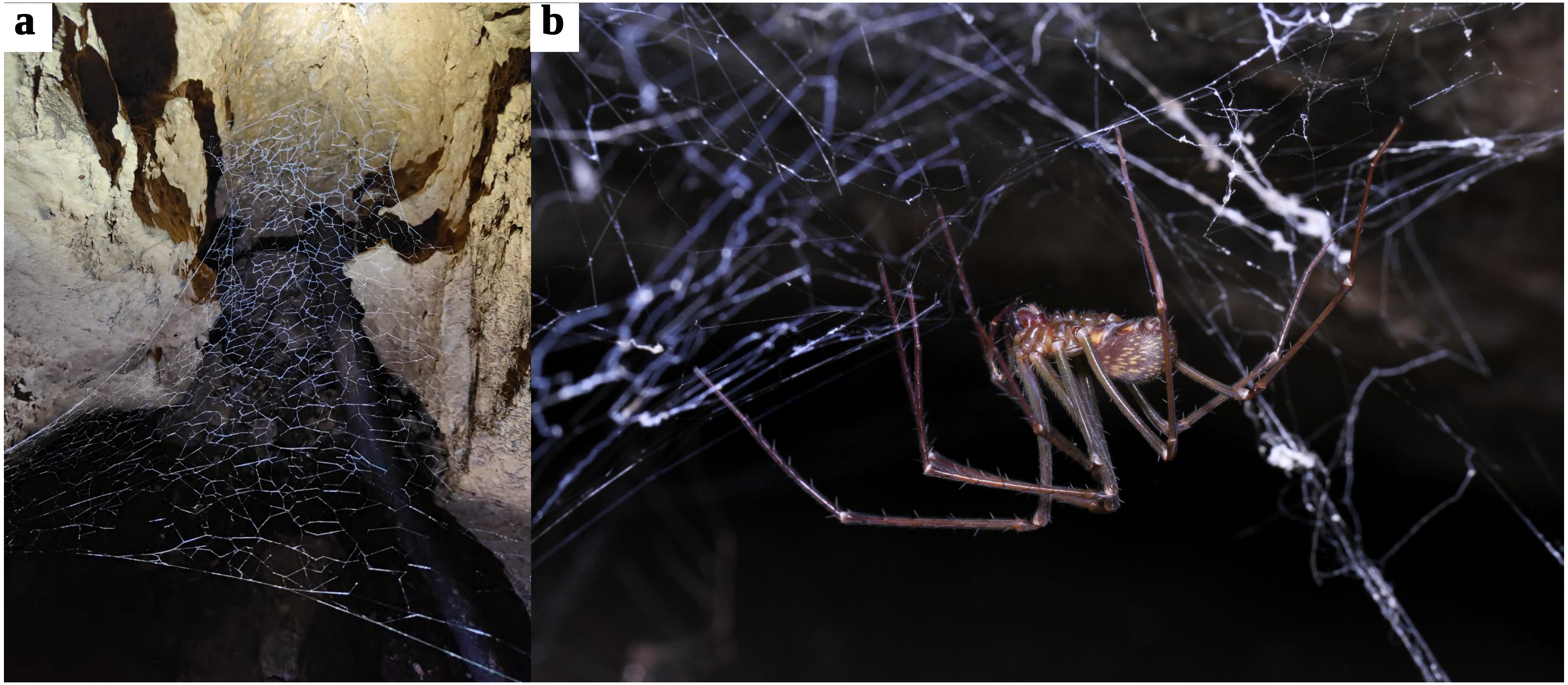
a
b

Supplementary Figure 1. The lampshade web spider *Ectatosticta davidi* and its web.
a: The web of lampshade web spider. b: A female lampshade web spider.

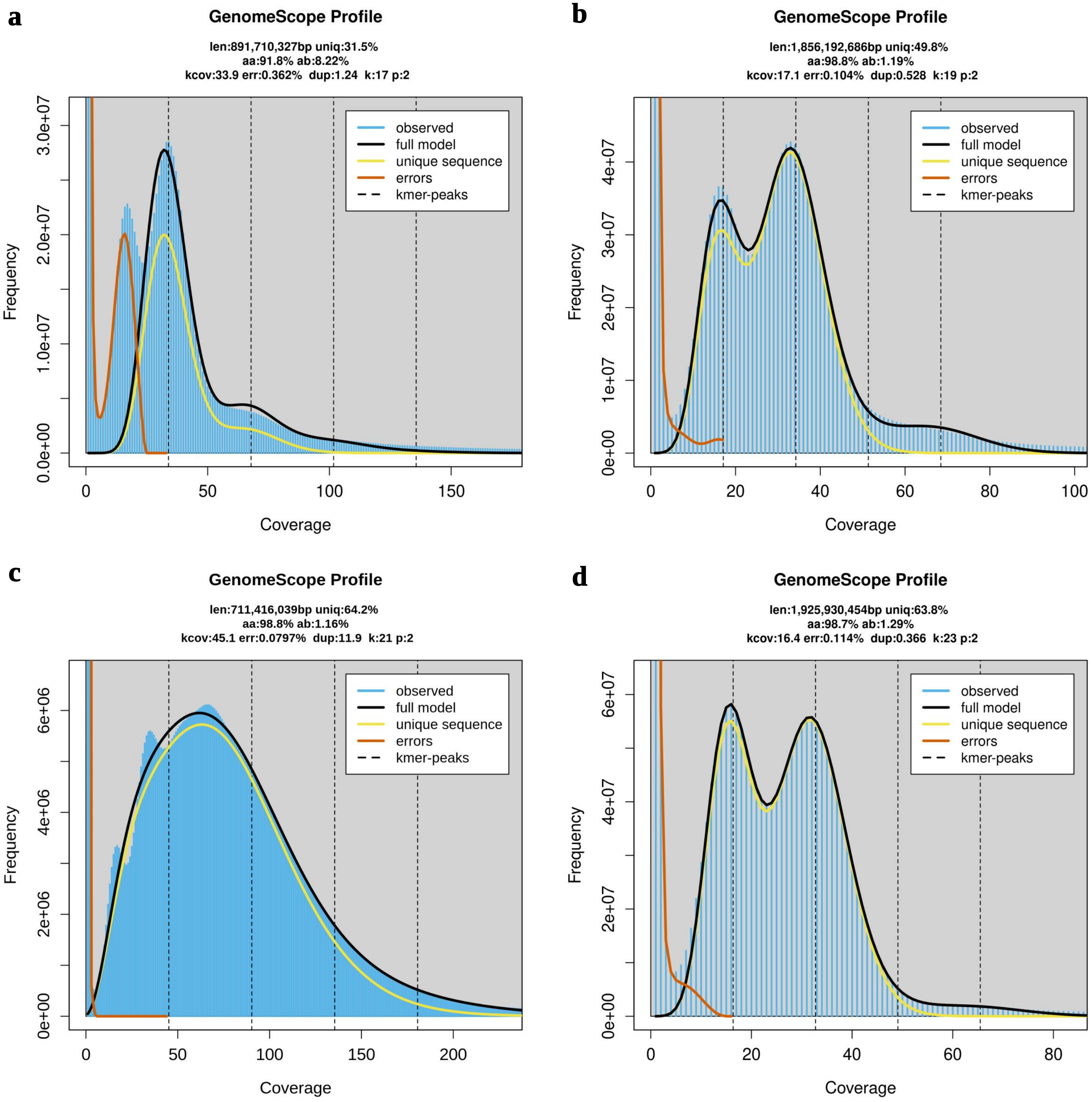

a
GenomeScope Profile
len:891,710,327bp uniq:31.5%
aa:91.8% ab:8.22%
kcov:33.9 err:0.362% dup:1.24 k:17 p:2
observed
full model
unique sequence
errors
kmer-peaks
Frequency
Coverage
b
GenomeScope Profile
len:1,856,192,686bp uniq:49.8%
aa:98.8% ab:1.19%
kcov:17.1 err:0.104% dup:0.528 k:19 p:2
c
GenomeScope Profile
len:711,416,039bp uniq:64.2%
aa:98.8% ab:1.16%
kcov:45.1 err:0.0797% dup:11.9 k:21 p:2
d
GenomeScope Profile
len:1,925,930,454bp uniq:63.8%
aa:98.7% ab:1.29%
kcov:16.4 err:0.114% dup:0.366 k:23 p:2

Supplementary Figure 2 . K-mer distribution of genome sequencing reads of *E. davidi.* K-mers (K = 17, 19, 21 and 23) were extracted from Illumina reads.

a: The peak 17-merdepth was 33.9, and the genome size was calculated as 891.7 Mb.

b: The peak 19-mer depthwas 17.1, and the genome size was calculated as 1,856.1 Mb.

c: The peak 21-mer depth was45.1, and the genome size was calculated as 711.4 Mb.

d: The peak 23-mer depth was 16.4,and the genome size was calculated as 1,925.9 Mb.

And the model fit better in 19-mer and 23-mer, and its estimated genome size of *E. davidi* is about 1,856.1 - 1,925.9 Mb， and the heterozygosity is 1.19% - 1.29%.

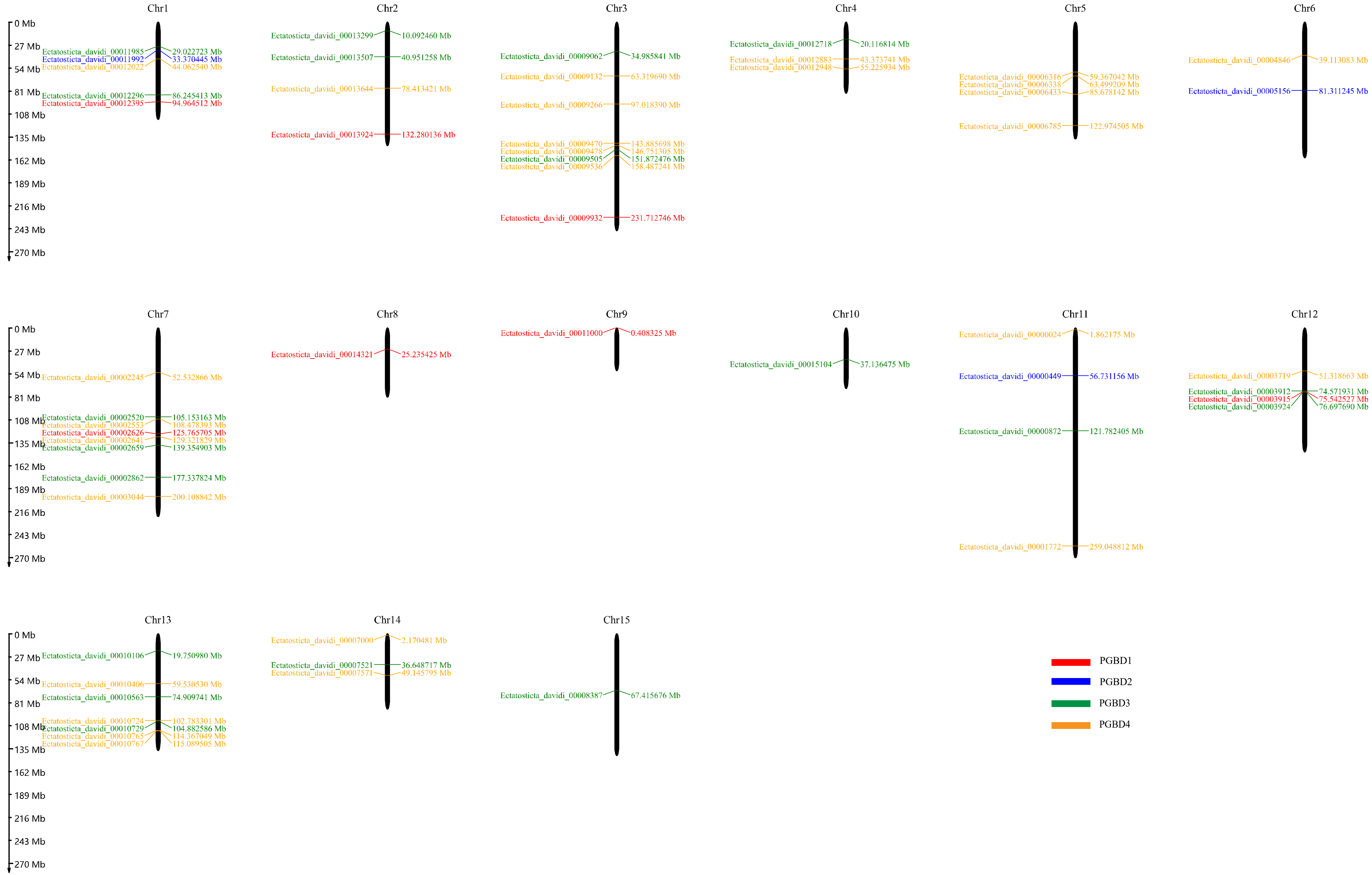
Chr1
Chr2
Chr3
Chr4
Chr5
Chr6
0 Mb
27 Mb
54 Mb
81 Mb
108 Mb
135 Mb
162 Mb
189 Mb
216 Mb
243 Mb
270 Mb
Ectatosticta_davidi_00011985 29.022723 Mb
Ectatosticta_davidi_00011992 33.370445 Mb
Ectatosticta_davidi_00012022 44.062540 Mb
Ectatosticta_davidi_00012296 86.245413 Mb
Ectatosticta_davidi_00012395 94.964512 Mb
Ectatosticta_davidi_00013299 10.092460 Mb
Ectatosticta_davidi_00013507 40.951258 Mb
Ectatosticta_davidi_00013644 78.413421 Mb
Ectatosticta_davidi_00013924 132.280136 Mb
Ectatosticta_davidi_00009062 34.985841 Mb
Ectatosticta_davidi_00009132 63.319690 Mb
Ectatosticta_davidi_00009266 97.018390 Mb
Ectatosticta_davidi_00009470 143.885698 Mb
Ectatosticta_davidi_00009478 146.751305 Mb
Ectatosticta_davidi_00009505 151.872476 Mb
Ectatosticta_davidi_00009536 158.487241 Mb
Ectatosticta_davidi_00009932 231.712746 Mb
Ectatosticta_davidi_00012718 20.116814 Mb
Ectatosticta_davidi_00012883 43.373741 Mb
Ectatosticta_davidi_00012948 55.225934 Mb
Ectatosticta_davidi_00006316 59.367042 Mb
Ectatosticta_davidi_00006338 63.499209 Mb
Ectatosticta_davidi_00006433 85.678142 Mb
Ectatosticta_davidi_00006785 122.974505 Mb
Ectatosticta_davidi_00004846 39.113083 Mb
Ectatosticta_davidi_00005156 81.311245 Mb
Chr7
Chr8
Chr9
Chr10
Chr11
Chr12
0 Mb
27 Mb
54 Mb
81 Mb
108 Mb
135 Mb
162 Mb
189 Mb
216 Mb
243 Mb
270 Mb
Ectatosticta_davidi_00002245 52.532866 Mb
Ectatosticta_davidi_00002520 105.153163 Mb
Ectatosticta_davidi_00002553 108.478393 Mb
Ectatosticta_davidi_00002626 125.765705 Mb
Ectatosticta_davidi_00002641 129.321829 Mb
Ectatosticta_davidi_00002659 139.354903 Mb
Ectatosticta_davidi_00002862 177.337824 Mb
Ectatosticta_davidi_00003044 200.108842 Mb
Ectatosticta_davidi_00014321 25.235425 Mb
Ectatosticta_davidi_00011000 0.408325 Mb
Ectatosticta_davidi_00015104 37.136475 Mb
Ectatosticta_davidi_00000024 1.862175 Mb
Ectatosticta_davidi_00000449 56.731156 Mb
Ectatosticta_davidi_00000872 121.782405 Mb
Ectatosticta_davidi_00001772 259.048812 Mb
Ectatosticta_davidi_00003719 51.318663 Mb
Ectatosticta_davidi_00003912 74.571931 Mb
Ectatosticta_davidi_00003915 75.542527 Mb
Ectatosticta_davidi_00003924 76.697690 Mb
Chr13
Chr14
Chr15
0 Mb
27 Mb
54 Mb
81 Mb
108 Mb
135 Mb
162 Mb
189 Mb
216 Mb
243 Mb
270 Mb
Ectatosticta_davidi_00010106 19.750980 Mb
Ectatosticta_davidi_00010406 59.530530 Mb
Ectatosticta_davidi_00010563 74.909741 Mb
Ectatosticta_davidi_00010724 102.783301 Mb
Ectatosticta_davidi_00010729 104.882586 Mb
Ectatosticta_davidi_00010765 114.367049 Mb
Ectatosticta_davidi_00010767 115.089505 Mb
Ectatosticta_davidi_00007000 2.170481 Mb
Ectatosticta_davidi_00007521 36.648717 Mb
Ectatosticta_davidi_00007571 49.145795 Mb
Ectatosticta_davidi_00008387 67.415676 Mb
PGBD1
PGBD2
PGBD3
PGBD4

Supplementary Figure 3. Position of *piggyBac* genes in the *E. davidi* genome.

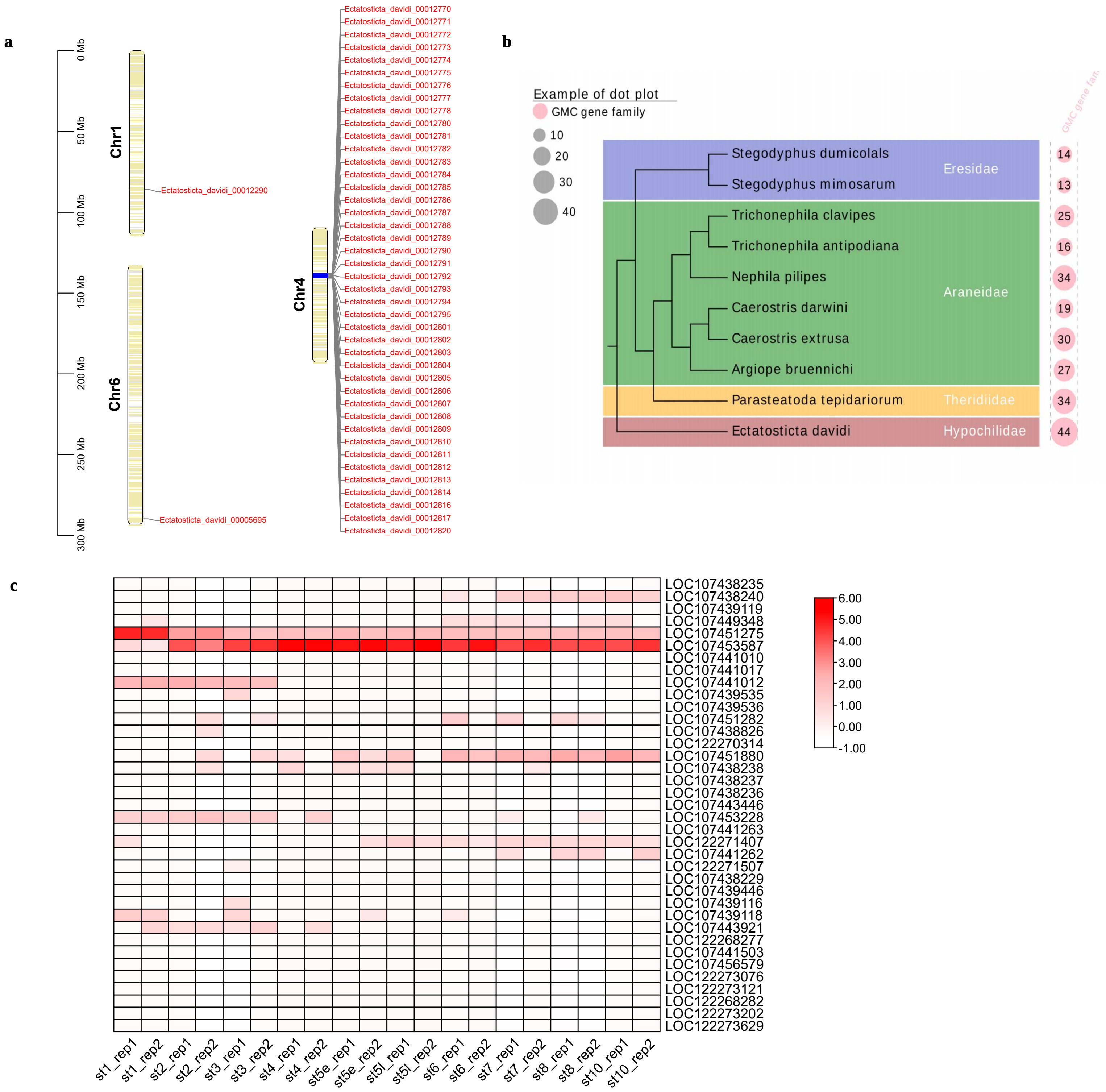
a
0 Mb
50 Mb
100 Mb
150 Mb
200 Mb
250 Mb
300 Mb
Chr1
Chr6
Chr4
Ectatosticta_davidi_00012290
Ectatosticta_davidi_00005695
Ectatosticta_davidi_00012770
Ectatosticta_davidi_00012771
Ectatosticta_davidi_00012772
Ectatosticta_davidi_00012773
Ectatosticta_davidi_00012774
Ectatosticta_davidi_00012775
Ectatosticta_davidi_00012776
Ectatosticta_davidi_00012777
Ectatosticta_davidi_00012778
Ectatosticta_davidi_00012780
Ectatosticta_davidi_00012781
Ectatosticta_davidi_00012782
Ectatosticta_davidi_00012783
Ectatosticta_davidi_00012784
Ectatosticta_davidi_00012785
Ectatosticta_davidi_00012786
Ectatosticta_davidi_00012787
Ectatosticta_davidi_00012788
Ectatosticta_davidi_00012789
Ectatosticta_davidi_00012790
Ectatosticta_davidi_00012791
Ectatosticta_davidi_00012792
Ectatosticta_davidi_00012793
Ectatosticta_davidi_00012794
Ectatosticta_davidi_00012795
Ectatosticta_davidi_00012801
Ectatosticta_davidi_00012802
Ectatosticta_davidi_00012803
Ectatosticta_davidi_00012804
Ectatosticta_davidi_00012805
Ectatosticta_davidi_00012806
Ectatosticta_davidi_00012807
Ectatosticta_davidi_00012808
Ectatosticta_davidi_00012809
Ectatosticta_davidi_00012810
Ectatosticta_davidi_00012811
Ectatosticta_davidi_00012812
Ectatosticta_davidi_00012813
Ectatosticta_davidi_00012814
Ectatosticta_davidi_00012816
Ectatosticta_davidi_00012817
Ectatosticta_davidi_00012820
b
Example of dot plot
GMC gene family
10
20
30
40
Stegodyphus dumicolals
Stegodyphus mimosarum
Eresidae
Trichonephila clavipes
Trichonephila antipodiana
Nephila pilipes
Caerostris darwini
Caerostris extrusa
Argiope bruennichi
Araneidae
Parasteatoda tepidariorum
Theridiidae
Ectatosticta davidi
Hypochilidae
14
13
25
16
34
19
30
27
34
44
c
LOC107438235
LOC107438240
LOC107439119
LOC107449348
LOC107451275
LOC107453587
LOC107441010
LOC107441017
LOC107441012
LOC107439535
LOC107439536
LOC107451282
LOC107438826
LOC122270314
LOC107451880
LOC107438238
LOC107438237
LOC107438236
LOC107443446
LOC107453228
LOC107441263
LOC122271407
LOC107441262
LOC122271507
LOC107438229
LOC107439446
LOC107439116
LOC107439118
LOC107443921
LOC122268277
LOC107441503
LOC107456579
LOC122273076
LOC122273121
LOC122268282
LOC122273202
LOC122273629
st1_rep1
st1_rep2
st2_rep1
st2_rep2
st3_rep1
st3_rep2
st4_rep1
st4_rep2
st5e_rep1
st5e_rep2
st5l_rep1
st5l_rep2
st6_rep1
st6_rep2
st7_rep1
st7_rep2
st8_rep1
st8_rep2
st10_rep1
st10_rep2
6.00
5.00
4.00
3.00
2.00
1.00
0.00
-1.00

Supplementary Figure 4. Comparative analysis of the GMC gene family in the *E. davidi* genome.

a: Position of the GMC gene family in the *E. davidi* genome.

b: Number of GMC gene family members in *E. davidi* and other spiders.

c: Transcriptome analysis of GMC genes in the spider *P. epidariorum* among different stages (stages 1-10).

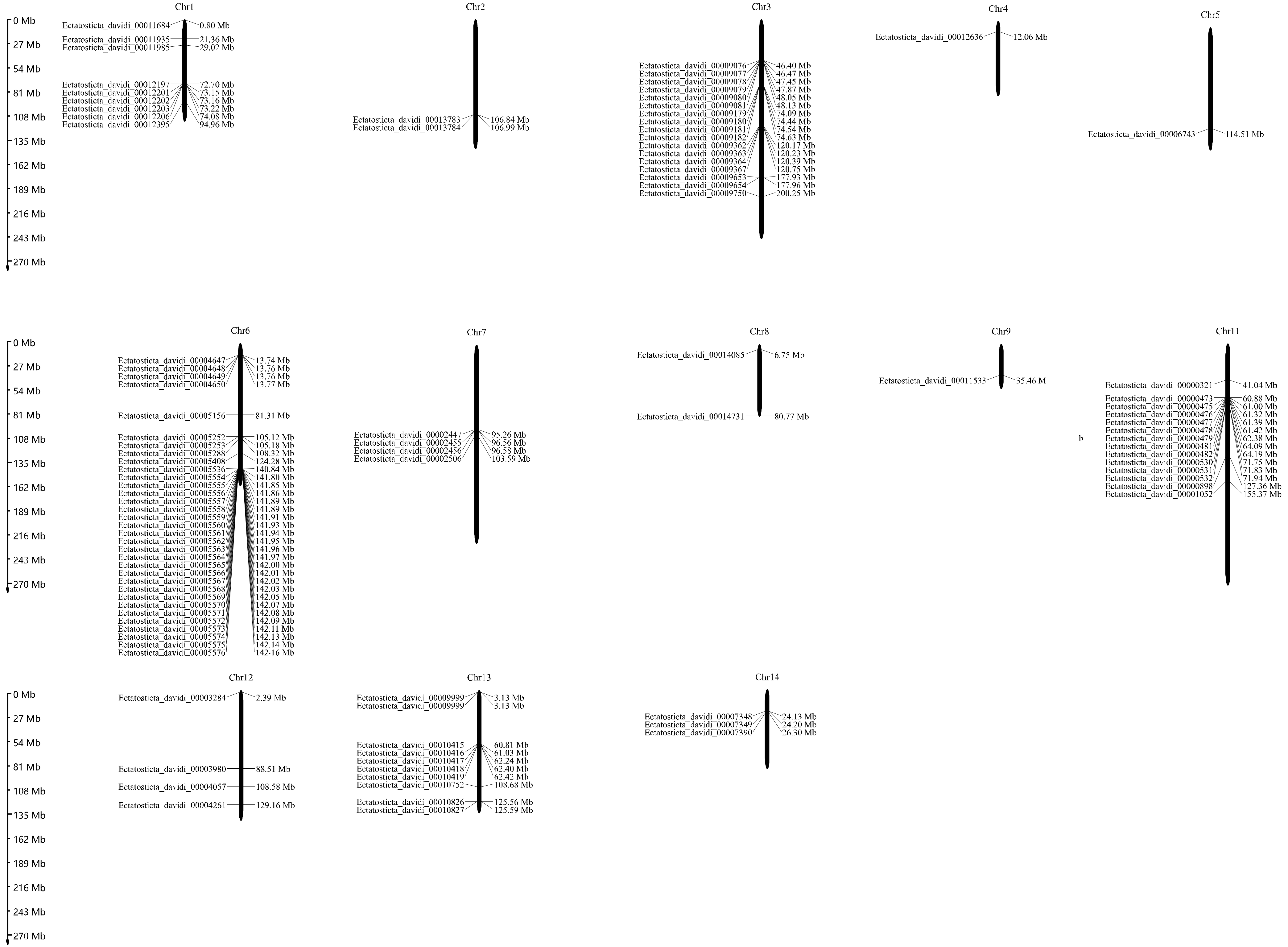

0 Mb
27 Mb
54 Mb
81 Mb
108 Mb
135 Mb
162 Mb
189 Mb
216 Mb
243 Mb
270 Mb
Chr1
Ectatosticta_davidi_00011684 0.80 Mb
Ectatosticta_davidi_00011935 21.36 Mb
Ectatosticta_davidi_00011985 29.02 Mb
Ectatosticta_davidi_00012197 72.70 Mb
Ectatosticta_davidi_00012201 73.15 Mb
Ectatosticta_davidi_00012202 73.16 Mb
Ectatosticta_davidi_00012203 73.22 Mb
Ectatosticta_davidi_00012206 74.08 Mb
Ectatosticta_davidi_00012395 94.96 Mb
Chr2
Ectatosticta_davidi_00013783 106.84 Mb
Ectatosticta_davidi_00013784 106.99 Mb
Chr3
Ectatosticta_davidi_00009076 46.40 Mb
Ectatosticta_davidi_00009077 46.47 Mb
Ectatosticta_davidi_00009078 47.45 Mb
Ectatosticta_davidi_00009079 47.87 Mb
Ectatosticta_davidi_00009080 48.05 Mb
Ectatosticta_davidi_00009081 48.13 Mb
Ectatosticta_davidi_00009179 74.09 Mb
Ectatosticta_davidi_00009180 74.44 Mb
Ectatosticta_davidi_00009181 74.54 Mb
Ectatosticta_davidi_00009182 74.63 Mb
Ectatosticta_davidi_00009362 120.17 Mb
Ectatosticta_davidi_00009363 120.23 Mb
Ectatosticta_davidi_00009364 120.39 Mb
Ectatosticta_davidi_00009367 120.75 Mb
Ectatosticta_davidi_00009653 177.93 Mb
Ectatosticta_davidi_00009654 177.96 Mb
Ectatosticta_davidi_00009750 200.25 Mb
Chr4
Ectatosticta_davidi_00012636 12.06 Mb
Chr5
Ectatosticta_davidi_00006743 114.51 Mb
Chr6
Ectatosticta_davidi_00004647 13.74 Mb
Ectatosticta_davidi_00004648 13.76 Mb
Ectatosticta_davidi_00004649 13.76 Mb
Ectatosticta_davidi_00004650 13.77 Mb
Ectatosticta_davidi_00005156 81.31 Mb
Ectatosticta_davidi_00005252 105.12 Mb
Ectatosticta_davidi_00005253 105.18 Mb
Ectatosticta_davidi_00005288 108.32 Mb
Ectatosticta_davidi_00005408 124.28 Mb
Ectatosticta_davidi_00005536 140.84 Mb
Ectatosticta_davidi_00005554 141.80 Mb
Ectatosticta_davidi_00005555 141.85 Mb
Ectatosticta_davidi_00005556 141.86 Mb
Ectatosticta_davidi_00005557 141.89 Mb
Ectatosticta_davidi_00005558 141.89 Mb
Ectatosticta_davidi_00005559 141.91 Mb
Ectatosticta_davidi_00005560 141.93 Mb
Ectatosticta_davidi_00005561 141.94 Mb
Ectatosticta_davidi_00005562 141.95 Mb
Ectatosticta_davidi_00005563 141.96 Mb
Ectatosticta_davidi_00005564 141.97 Mb
Ectatosticta_davidi_00005565 142.00 Mb
Ectatosticta_davidi_00005566 142.01 Mb
Ectatosticta_davidi_00005567 142.02 Mb
Ectatosticta_davidi_00005568 142.03 Mb
Ectatosticta_davidi_00005569 142.05 Mb
Ectatosticta_davidi_00005570 142.07 Mb
Ectatosticta_davidi_00005571 142.08 Mb
Ectatosticta_davidi_00005572 142.09 Mb
Ectatosticta_davidi_00005573 142.11 Mb
Ectatosticta_davidi_00005574 142.13 Mb
Ectatosticta_davidi_00005575 142.14 Mb
Ectatosticta_davidi_00005576 142.16 Mb
Chr7
Ectatosticta_davidi_00002447 95.26 Mb
Ectatosticta_davidi_00002455 96.56 Mb
Ectatosticta_davidi_00002456 96.58 Mb
Ectatosticta_davidi_00002506 103.59 Mb
Chr8
Ectatosticta_davidi_00014085 6.75 Mb
Ectatosticta_davidi_00014731 80.77 Mb
Chr9
Ectatosticta_davidi_00011533 35.46 M
b
Chr11
Ectatosticta_davidi_00000321 41.04 Mb
Ectatosticta_davidi_00000473 60.88 Mb
Ectatosticta_davidi_00000475 61.00 Mb
Ectatosticta_davidi_00000476 61.32 Mb
Ectatosticta_davidi_00000477 61.39 Mb
Ectatosticta_davidi_00000478 61.42 Mb
Ectatosticta_davidi_00000479 62.38 Mb
Ectatosticta_davidi_00000481 64.09 Mb
Ectatosticta_davidi_00000482 64.19 Mb
Ectatosticta_davidi_00000530 71.75 Mb
Ectatosticta_davidi_00000531 71.83 Mb
Ectatosticta_davidi_00000532 71.94 Mb
Ectatosticta_davidi_00000898 127.36 Mb
Ectatosticta_davidi_00001052 155.37 Mb
Chr12
Ectatosticta_davidi_00003284 2.39 Mb
Ectatosticta_davidi_00003980 88.51 Mb
Ectatosticta_davidi_00004057 108.58 Mb
Ectatosticta_davidi_00004261 129.16 Mb
Chr13
Ectatosticta_davidi_00009999 3.13 Mb
Ectatosticta_davidi_00009999 3.13 Mb
Ectatosticta_davidi_00010415 60.81 Mb
Ectatosticta_davidi_00010416 61.03 Mb
Ectatosticta_davidi_00010417 62.24 Mb
Ectatosticta_davidi_00010418 62.40 Mb
Ectatosticta_davidi_00010419 62.42 Mb
Ectatosticta_davidi_00010752 108.68 Mb
Ectatosticta_davidi_00010826 125.56 Mb
Ectatosticta_davidi_00010827 125.59 Mb
Chr14
Ectatosticta_davidi_00007348 24.13 Mb
Ectatosticta_davidi_00007349 24.20 Mb
Ectatosticta_davidi_00007390 26.30 Mb

Supplementary Figure 5 . Position of IR/iGluR genes in the *E. davidi* genome.

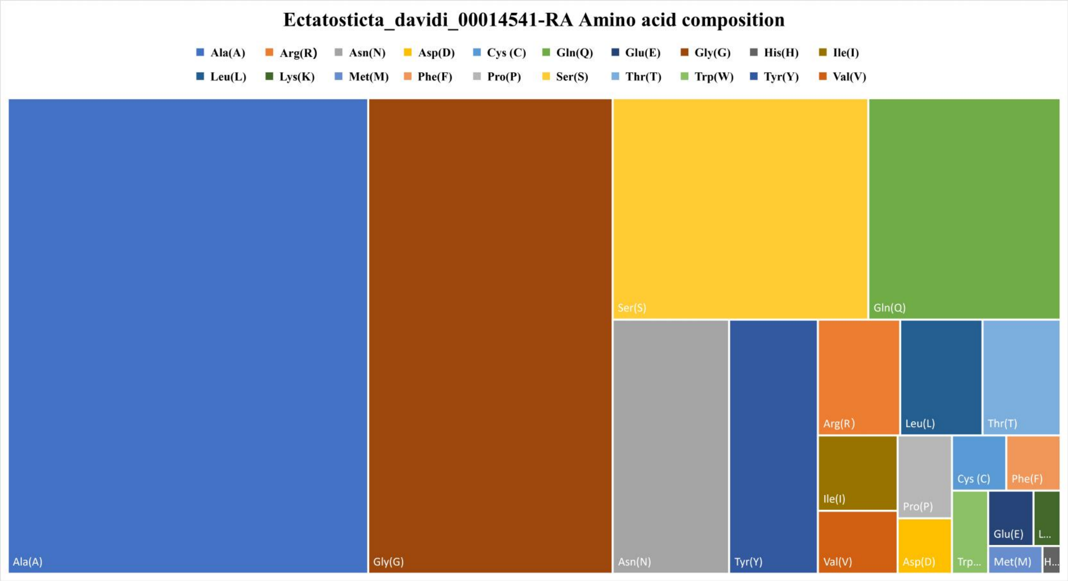
Ectatosticta_davidi_00014541-RA Amino acid composition
Ala(A) Arg(R) Asn(N) Asp(D) Cys (C) Gln(Q) Glu(E) Gly(G) His(H) Ile(I)
Leu(L) Lys(K) Met(M) Phe(F) Pro(P) Ser(S) Thr(T) Trp(W) Tyr(Y) Val(V)
Ala(A)
Gly(G)
Ser(S)
Gln(Q)
Asn(N)
Tyr(Y)
Arg(R)
Leu(L)
Thr(T)
Ile(I)
Pro(P)
Cys (C)
Phe(F)
Val(V)
Asp(D)
Trp...
Glu(E)
L...
Met(M)
H...

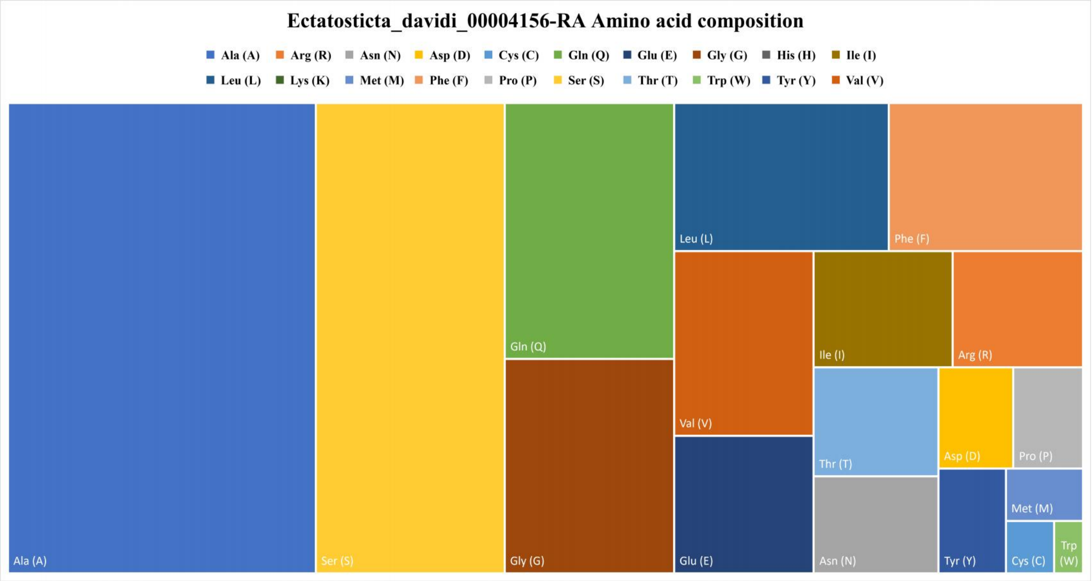
Ectatosticta_davidi_00004156-RA Amino acid composition
Ala (A) Arg (R) Asn (N) Asp (D) Cys (C) Gln (Q) Glu (E) Gly (G) His (H) Ile (I)
Leu (L) Lys (K) Met (M) Phe (F) Pro (P) Ser (S) Thr (T) Trp (W) Tyr (Y) Val (V)
Ala (A)
Ser (S)
Gln (Q)
Gly (G)
Leu (L)
Phe (F)
Val (V)
Ile (I)
Arg (R)
Glu (E)
Thr (T)
Asp (D)
Pro (P)
Asn (N)
Tyr (Y)
Met (M)
Cys (C)
Trp (W)

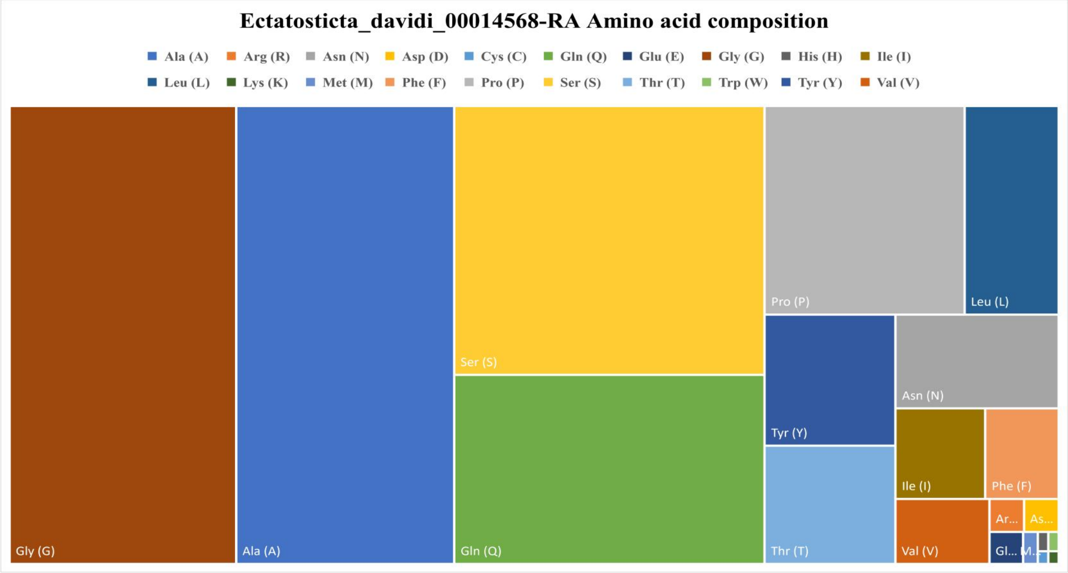
Ectatosticta_davidi_00014568-RA Amino acid composition
Ala (A) Arg (R) Asn (N) Asp (D) Cys (C) Gln (Q) Glu (E) Gly (G) His (H) Ile (I)
Leu (L) Lys (K) Met (M) Phe (F) Pro (P) Ser (S) Thr (T) Trp (W) Tyr (Y) Val (V)
Gly (G)
Ala (A)
Ser (S)
Gln (Q)
Pro (P)
Leu (L)
Tyr (Y)
Asn (N)
Thr (T)
Ile (I)
Phe (F)
Val (V)
Ar...
As...
Gl...
M...

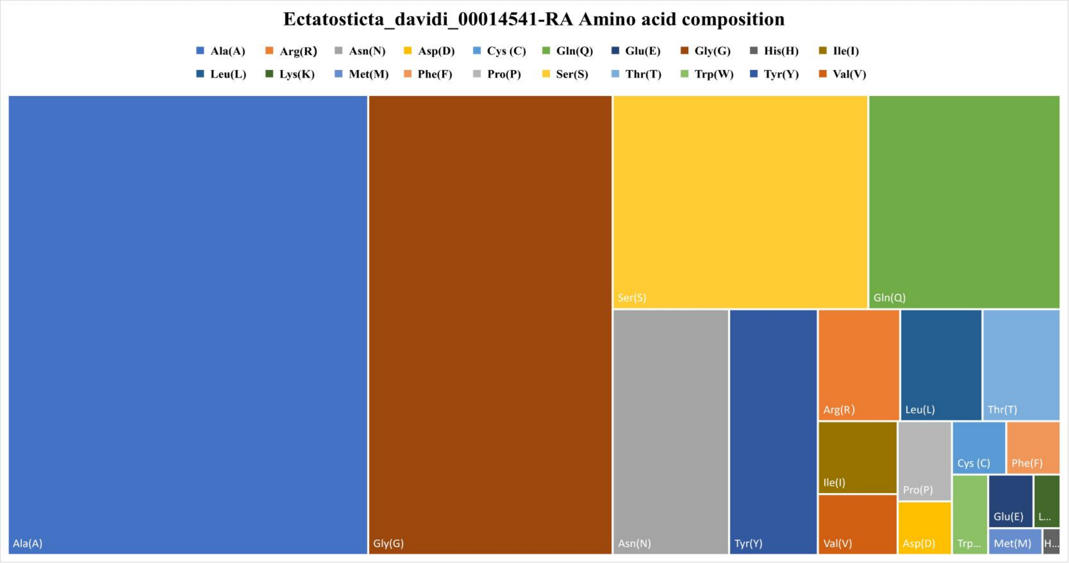
Ectatosticta_davidi_00014541-RA Amino acid composition
Ala(A) Arg(R) Asn(N) Asp(D) Cys (C) Gln(Q) Glu(E) Gly(G) His(H) Ile(I)
Leu(L) Lys(K) Met(M) Phe(F) Pro(P) Ser(S) Thr(T) Trp(W) Tyr(Y) Val(V)
Ala(A)
Gly(G)
Ser(S)
Gln(Q)
Asn(N)
Tyr(Y)
Arg(R)
Leu(L)
Thr(T)
Ile(I)
Pro(P)
Cys (C)
Phe(F)
Val(V)
Asp(D)
Trp...
Glu(E)
L...
Met(M)
H...

Supplementary Figure 6 . Amino acid composition of spider silk protein in *E. davidi.*

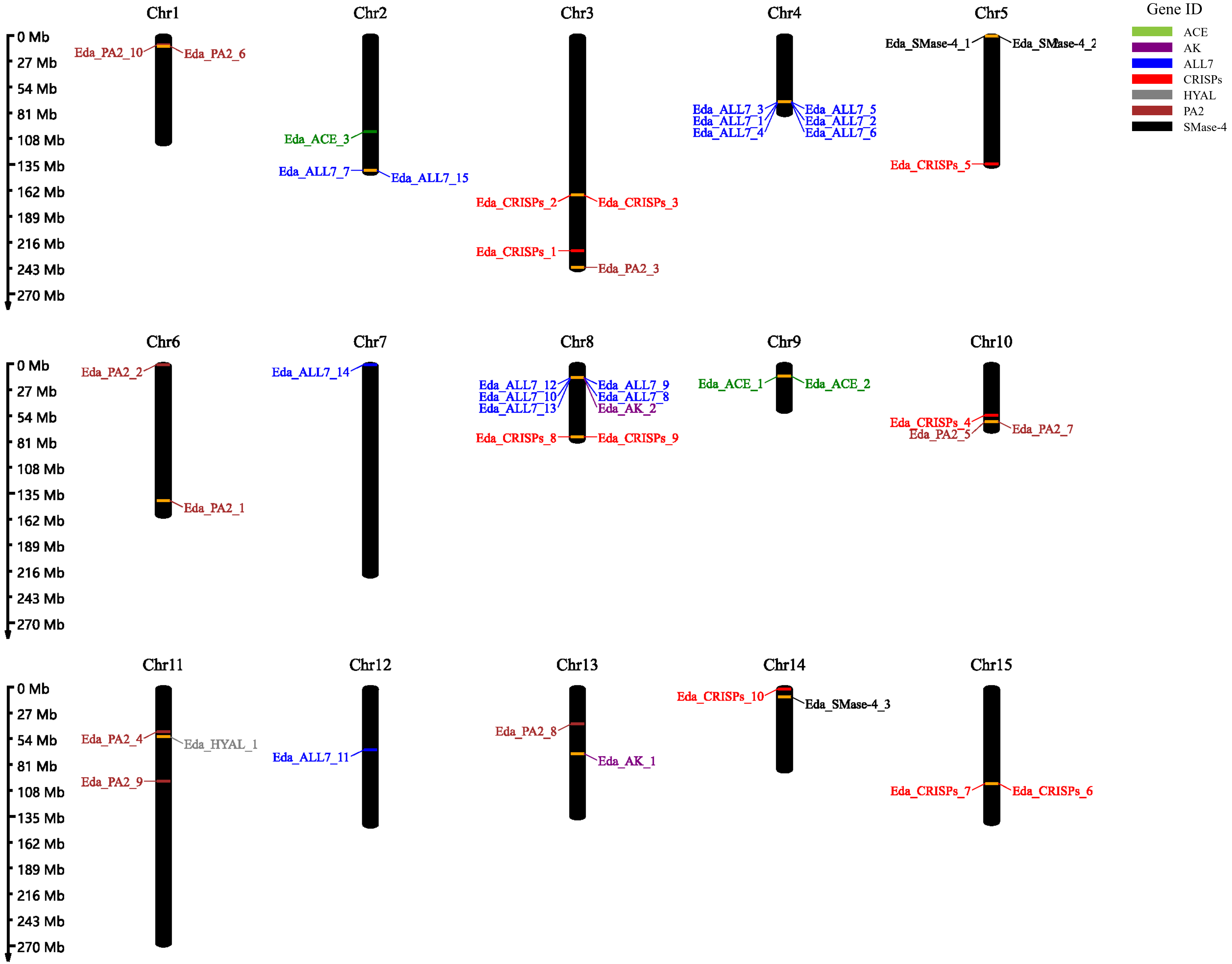

Chr1
Chr2
Chr3
Chr4
Chr5
Chr6
Chr7
Chr8
Chr9
Chr10
Chr11
Chr12
Chr13
Chr14
Chr15
0 Mb
27 Mb
54 Mb
81 Mb
108 Mb
135 Mb
162 Mb
189 Mb
216 Mb
243 Mb
270 Mb
Gene ID
ACE
AK
ALL7
CRISPs
HYAL
PA2
SMase-4
Eda_PA2_10
Eda_PA2_6
Eda_ACE_3
Eda_ALL7_7
Eda_ALL7_15
Eda_CRISPs_2
Eda_CRISPs_3
Eda_CRISPs_1
Eda_PA2_3
Eda_ALL7_3
Eda_ALL7_1
Eda_ALL7_4
Eda_ALL7_5
Eda_ALL7_2
Eda_ALL7_6
Eda_SMase-4_1
Eda_SMase-4_2
Eda_CRISPs_5
Eda_PA2_2
Eda_PA2_1
Eda_ALL7_14
Eda_ALL7_12
Eda_ALL7_10
Eda_ALL7_13
Eda_ALL7_9
Eda_ALL7_8
Eda_AK_2
Eda_CRISPs_8
Eda_CRISPs_9
Eda_ACE_1
Eda_ACE_2
Eda_CRISPs_4
Eda_PA2_5
Eda_PA2_7
Eda_PA2_4
Eda_HYAL_1
Eda_PA2_9
Eda_ALL7_11
Eda_PA2_8
Eda_AK_1
Eda_CRISPs_10
Eda_SMase-4_3
Eda_CRISPs_7
Eda_CRISPs_6

Supplementary Figure 7. Position of toxin genes in the *E. davidi* genome.

Supplementary Table 1. Assembly statistics of *E. davidi* and other 29 public online spider species.

number	Family	Species	Genome size (Gb)	Scaffold N50 (kbp)	Contig N50 (kbp)	Accession number	Genome level
1	Hypochilidae	*Ectatosticta davidi*	2.16	146,182.00	579.23	this article	Chromosome
2	Araneidae	*Araneus ventricosus*	3.65	59.62	59.62	BGPR01000001-BGPR01300721	Scaffold
3	Araneidae	*Argiope bruennichi*	1.67	124,236.00	284.77	GCA_015342795.1	Chromosome
4	Araneidae	*Argiope aurantia*	1.84	266.79	34.41	GCA_026543865.1	Scaffold
5	Araneidae	*Argiope trifasciata*	1.88	1,425.81	35.47	GCA_026543055.1	Scaffold
6	Araneidae	*Caerostris darwini*	1.5	440.87	330.29	GCA_021605075.1	Scaffold
7	Araneidae	*Caerostris extrusa*	1.42	98.47	98.43	GCA_021605095.1	Scaffold
8	Araneidae	*Nephila pilipes*	2.69	292.14	41.84	GCA_019974015.1	Scaffold
9	Araneidae	*Trichonephila antipodiana*	2.29	172,892.00	1,138.00	http://dx.doi.org/10.5524/100868	Chromosome
10	Araneidae	*Trichonephila clavipes*	2.44	62.96	7.99	GCA_002102615.1	Scaffold
11	Araneidae	*Trichonephila clavata*	2.49	112.99	99.66	GCA_019973975.1	Scaffold
12	Araneidae	*Trichonephila inaurata*	2,50	28.26	37.55	GCA_019973955.1	Scaffold
13	Drymusidae	*Loxosceles reclusa*	3.26	63.24	1.83	GCA_001188405.1	Scaffold
14	Dysderidae	*Dysdera silvatica*	1.36	38.02	25.71	GCA_006491805.1	Scaffold
15	Eresidae	*Stegodyphus dumicola*	2.55	254.13	254.13	GCA_010614865.1	Scaffold
16	Eresidae	*Stegodyphus mimosarum*	2.74	480.64	40.15	GCA_000611955.2	Scaffold
17	Tetragnathidae	*Meta bourneti*	1.38	104,066.34	8,589.33	GCA_933210815.1	Chromosome
18	Tetragnathidae	*Metellina segmentata*	1.66	129,427.59	3,433.00	GCA_947359465.1	Chromosome
19	Tetragnathidae	*Tetragnatha kauaiensis*	1.08	1,906.25	8,246.00	https://doi.org/10.5061/dryad.b2rbnzsgr	Scaffold
20	Tetragnathidae	*Tetragnatha versicolor*	1.04	55,124.72	7,638.61	GCA_024610695.1	Scaffold
21	Theraphosidae	*Acanthoscurria geniculata*	7.18	20.29	0.54	GCA_000661875.1	Scaffold
22	Theridiidae	*Latrodectus hesperus*	1.23	39.47	15.96	GCA_000697925.2	Scaffold
23	Theridiidae	*Latrodectus elegans*	1.57	114,313.28	4,335.61	http://doi.org/10.5524/102210	Chromosome
24	Theridiidae	*Parasteatoda tepidariorum*	1.45	4,055.36	10.15	GCA_000365465.3	Scaffold
25	Theridiidae	*Anelosimus studiosus*	2.03	4.79	1.13	GCA_008297655.1	Scaffold
26	Linyphiidae	*Hylyphantes graminicola*	0.9	77,070	889.59	https:// doi.org/10.11922/ [illegible]	Chromosome
27	Linyphiidae	*Oedothorax gibbosus*	0.82	979.33	979.33	GCA_019343175.1	Chromosome
28	Lycosidae	*Pardosa pseudoannulata*	4.21	711.40	23.23	GCA_008065355.1	Scaffold
29	Uloboridae	*Uloborus diversus*	2.15	185,519.77	452.78	GCA_026930045.1	Chromosome
30	Pisauridae	*Dolomedes plantarius*	2.78	216,720.62	2,276.77	GCA_907164885.1	Chromosome

Supplementary Table 2. Summary of each step-in construction of the *E. davidi* genome assembly.

Assembly	Total length (Gb)	No. scaffolds (chromosome)	Scaffold N50 length (Kb)	Longest scaffold (MB)	GC (%)	BUSCO (n = 1,013) (%)			
						C	D	F	M
Raven	2.37	6,654	593	5.54	36	95.6	8.7	3	1.4
Purge haplotigs	2.16	4,702	656	5.54	36	94	5.4	3.3	2.7
Nextpolish	2.16	4,702	658	5.58	36	95	6	2.7	2.3
Hi-C	2.16	2,955(15)	146,184	272.65	36	95.5	4.6	2.4	2.1
Final assembly	2.16	1,600(15)	146,182	272.65	36	95.4	4.6	2.4	2.2

Supplementary Table 3. Statistics of the repetitive sequences identified in *E. davidi.*

Type	Number of elements	Length (bp)	% of genome
**SINEs:**	**292,875**	**54,975,840**	**2.54**
MIR	194,484	39,222,618	1.81
tRNA-Deu-L2	61,428	10,671,046	0.49
tRNA-V-CR1	26,598	3,101,854	0.14
**LINEs:**	**599,905**	**231,673,380**	**10.69**
Penelope	329,682	119,882,994	5.53
L2/CR1/Rex	140,458	49,187,444	2.27
R1/LOA/Jockey	73,705	41,076,677	1.9
**LTRs:**	**101,680**	**46,722,745**	**2.16**
Gypsy/DIRS1	62,337	34,814,061	1.61
Ty1/Copia	23,848	6,267,005	0.29
BEL/Pao	4,918	3,215,070	0.15
**DNA transposons:**	**892,773**	**234,677,934**	**10.83**
hobo-Activator	561,939	145,427,835	6.71
Rolling-circles	269,792	54,887,904	2.53
Tc1-IS630-Pogo	83,907	25,015,895	1.15
PiggyBac	9,686	3,542,450	0.16
**Unclassified**	**3,799,221**	**784,525,440**	**36.21**
**Total interspersed repeats**	**-**	**1,352,575,339**	**62.43**
**Small RNA**	**60,862**	**11,869,051**	**0.55**
**Satellites**	**10,197**	**3,734,867**	**0.17**
**Simple repeats**	**318,310**	**21,505,201**	**0.99**
**Low complexity**	**34603**	**1795476**	**0.08**

Supplementary Table 4. Protein sequences used for identifying the homologous toxin *piggyBac* gene families.

Database	Accession Number	Species	Protein name
NCBI	XP_028147889.1	*Diabrotica virgifera virgifera*	piggyBac transposable element-derived protein 4-like
NCBI	XP_029175030.1	*Nylanderia fulva*	piggyBac transposable element-derived protein 4-like
NCBI	XP_032682009.1	*Odontomachus brunneus*	piggyBac transposable element-derived protein 4-like
NCBI	XP_028152851.1	*Diabrotica virgifera virgifera*	piggyBac transposable element-derived protein 2-like
NCBI	XP_028150670.1	*Diabrotica virgifera virgifera*	piggyBac transposable element-derived protein 3-like
NCBI	XP_028149591.1	*Diabrotica virgifera virgifera*	piggyBac transposable element-derived protein 2-like
NCBI	XP_028145650.1	*Diabrotica virgifera virgifera*	piggyBac transposable element-derived protein 3-like
NCBI	XP_028140639.1	*Diabrotica virgifera virgifera*	piggyBac transposable element-derived protein 2-like
NCBI	XP_028139459.1	*Diabrotica virgifera virgifera*	piggyBac transposable element-derived protein 4-like
NCBI	XP_028137852.1	*Diabrotica virgifera virgifera*	piggyBac transposable element-derived protein 3-like
NCBI	XP_028136253.1	*Diabrotica virgifera virgifera*	piggyBac transposable element-derived protein 3-like
NCBI	XP_028135469.1	*Diabrotica virgifera virgifera*	piggyBac transposable element-derived protein 4-like
NCBI	XP_028134679.1	*Diabrotica virgifera virgifera*	piggyBac transposable element-derived protein 4-like
NCBI	XP_028134190.1	*Diabrotica virgifera virgifera*	piggyBac transposable element-derived protein 3-like
NCBI	XP_028133560.1	*Diabrotica virgifera virgifera*	piggyBac transposable element-derived protein 4-like
NCBI	XP_028133559.1	*Diabrotica virgifera virgifera*	piggyBac transposable element-derived protein 4-like
NCBI	XP_028133271.1	*Diabrotica virgifera virgifera*	piggyBac transposable element-derived protein 2-like
NCBI	XP_028131075.1	*Diabrotica virgifera virgifera*	piggyBac transposable element-derived protein 2-like
NCBI	XP_028155570.1	*Diabrotica virgifera virgifera*	piggyBac transposable element-derived protein 3-like
NCBI	XP_028142266.1	*Diabrotica virgifera virgifera*	piggyBac transposable element-derived protein 3-like
NCBI	XP_028149747.1	*Diabrotica virgifera virgifera*	piggyBac transposable element-derived protein 3-like, partial
NCBI	XP_028141310.1	*Diabrotica virgifera virgifera*	piggyBac transposable element-derived protein 3-like
NCBI	XP_028141006.1	*Diabrotica virgifera virgifera*	piggyBac transposable element-derived protein 3-like
NCBI	XP_028129758.1	*Diabrotica virgifera virgifera*	piggyBac transposable element-derived protein 3-like
NCBI	XP_028139824.1	*Diabrotica virgifera virgifera*	piggyBac transposable element-derived protein 2-like
NCBI	XP_014271535.1	*Halyomorpha halys*	piggyBac transposable element-derived protein 3
NCBI	XP_022910490.1	*Onthophagus taurus*	piggyBac transposable element-derived protein 4-like
NCBI	XP_032688514.1	*Odontomachus brunneus*	piggyBac transposable element-derived protein 3-like
NCBI	XP_032688641.1	*Odontomachus brunneus*	piggyBac transposable element-derived protein 4-like
NCBI	XP_032689124.1	*Odontomachus brunneus*	piggyBac transposable element-derived protein 3-like
NCBI	XP_032685599.1	*Odontomachus brunneus*	piggyBac transposable element-derived protein 4-like
NCBI	XP_032682010.1	*Odontomachus brunneus*	piggyBac transposable element-derived protein 4-like

NCBI	XP_032677064.1	*Odontomachus brunneus*	piggyBac transposable element-derived protein 4-like
NCBI	XP_032671956.1	*Odontomachus brunneus*	piggyBac transposable element-derived protein 4-like
NCBI	XP_029154909.1	*Nylanderia fulva*	piggyBac transposable element-derived protein 4-like
NCBI	XP_015124550.1	*Diachasma alloeum*	piggyBac transposable element-derived protein 4-like
NCBI	XP_015124497.1	*Diachasma alloeum*	piggyBac transposable element-derived protein 4-like
NCBI	XP_015122054.1	*Diachasma alloeum*	piggyBac transposable element-derived protein 4-like
NCBI	XP_015121722.1	*Diachasma alloeum*	piggyBac transposable element-derived protein 4-like
NCBI	XP_015121474.1	*Diachasma alloeum*	piggyBac transposable element-derived protein 4-like
NCBI	XP_015118406.1	*Diachasma alloeum*	piggyBac transposable element-derived protein 4-like
NCBI	XP_015113011.1	*Diachasma alloeum*	piggyBac transposable element-derived protein 3-like
NCBI	XP_015111779.1	*Diachasma alloeum*	piggyBac transposable element-derived protein 4-like
NCBI	XP_015120075.1	*Diachasma alloeum*	piggyBac transposable element-derived protein 4 isoform X1
NCBI	XP_015120086.1	*Diachasma alloeum*	piggyBac transposable element-derived protein 4 isoform X1
NCBI	XP_024219636.1	*Halyomorpha halys*	piggyBac transposable element-derived protein 4-like
NCBI	XP_024214119.1	*Halyomorpha halys*	piggyBac transposable element-derived protein 4-like
NCBI	XP_024214026.1	*Halyomorpha halys*	piggyBac transposable element-derived protein 4-like
NCBI	XP_024220084.1	*Halyomorpha halys*	piggyBac transposable element-derived protein 4-like
NCBI	XP_014290398.1	*Halyomorpha halys*	piggyBac transposable element-derived protein 4-like
NCBI	XP_014276908.1	*Halyomorpha halys*	piggyBac transposable element-derived protein 4-like
NCBI	XP_022914481.1	*Onthophagus taurus*	piggyBac transposable element-derived protein 4-like
NCBI	XP_022911139.1	*Onthophagus taurus*	piggyBac transposable element-derived protein 4-like
NCBI	XP_022913435.1	*Onthophagus taurus*	piggyBac transposable element-derived protein 4-like
NCBI	XP_022910938.1	*Onthophagus taurus*	piggyBac transposable element-derived protein 4-like
NCBI	XP_022910406.1	*Onthophagus taurus*	piggyBac transposable element-derived protein 3-like
NCBI	XP_022910826.1	*Onthophagus taurus*	piggyBac transposable element-derived protein 4-like

Supplementary Table 5. Extremely enpansion gene families of *E. davidi.*

Orthogroups	Number	Gene name
OG0000241	34	X-element\ORF2: Probable RNA-directed DNA polymerase from transposon X-
OG0000031	31	Ir93a: Ionotropic receptor 93a
OG0000409	29	Transposable element P transposase
OG0000025	28	Cuticle protein 16.8
OG0000359	27	Uncharacterized protein
OG0000395	23	PiggyBac transposable element-derived protein 4
OG0000813	18	PGBD3: PiggyBac transposable element-derived protein 3
OG0000640	20	Uncharacterized protein
OG0000375	19	CUL1: Cullin-1
OG0000991	19	DNA-directed DNA polymerase
OG0001036	19	Helitron_like_N domain-containing protein
OG0000175	11	Gld: Glucose dehydrogenase
OG0002245	10	CYP3A8: Cytochrome P450 3A8
OG0000559	8	CYP2J2: Cytochrome P450 2J2
OG0000435	18	Transposable element P transposase
OG0000567	18	uncharacterized protein
OG0001094	18	DSCAM: Down syndrome cell adhesion molecule
OG0000340	17	ANK2_11: Ankyrin-2
OG0000118	16	Transposable element P transposase
OG0000321	16	PGBD1: PiggyBac transposable element-derived protein 1
OG0000433	16	atk: Protein artichoke
OG0001563	16	PiggyBac transposable element-derived protein 4
OG0001565	16	Uncharacterized protein
OG0000785	15	SRRD: SRR1-like protein
OG0000180	14	Adult-specific rigid cuticular protein 11.9
OG0000182	14	Uncharacterized protein
OG0000250	14	thap1-b: THAP domain-containing protein 1 B
OG0001566	14	Transposable element P transposase
OG0002064	14	DNA transposase THAP9
OG0000115	13	Uncharacterized protein
OG0000562	13	Endonuclease
OG0001797	13	YqaJ domain-containing protein
OG0001227	12	Tl: Protein toll
OG0000667	11	uncharacterized protein
OG0002375	11	Uncharacterized protein

OG0000330	10	Clotting factor C
OG0001240	10	Ago2: Protein argonaute-2
OG0001788	10	Rpl14: 60S ribosomal protein L14
OG0000626	9	Uncharacterized protein
OG0000875	9	Endochitinase
OG0001028	9	Uncharacterized protein
OG0001532	9	anks6: Ankyrin repeat and SAM domain-containing protein 6
OG0001793	9	Putative p-14 hm
OG0002536	9	Uncharacterized protein
OG0003309	9	YqaJ domain-containing protei
OG0003310	9	Alkaline nuclease
OG0005064	9	spop: Speckle-type POZ protein
OG0000598	8	PNLIPRP3: Pancreatic lipase-related protein 3
OG0002241	8	Clotting factor B
OG0003771	8	PGBD4: PiggyBac transposable element-derived protein 4 (
OG0004751	8	Uncharacterized protein
OG0004853	7	THAP-type domain-containing protein
OG0002663	6	RTase: Probable RNA-directed DNA polymerase from transposon BS
OG0005887	6	SWIM-type domain-containing protein
OG0008529	6	Uncharacterized protein
OG0009887	6	Clone 988 transcribed RNA sequence (Plectreurys tristis)
OG0000148	5	Retrovirus-related Pol polyprotein from transposon 412
OG0001133	5	gdf-8: Growth/differentiation factor 8
OG0001941	5	Exd1: piRNA biogenesis protein EXD1
OG0002024	5	Uncharacterized protein
OG0002517	5	fam166b: Protein FAM166B
OG0006458	5	Uncharacterized protein
OG0007430	5	YqaJ domain-containing protein
OG0008644	5	Integrase_H2C2 domain-containing protein
OG0004168	4	U-scoloptoxin(01)-Cw1a
OG0009875	4	Uncharacterized protein

Supplementary Table 6. The name of *piggyBac* genes (PGBD) in *E. davidi.*

Gene ID	Name
Ectatosticta_davidi_00002626	PGBD1
Ectatosticta_davidi_00003915	PGBD1
Ectatosticta_davidi_00009932	PGBD1
Ectatosticta_davidi_00011000	PGBD1
Ectatosticta_davidi_00012395	PGBD1
Ectatosticta_davidi_00013924	PGBD1
Ectatosticta_davidi_00014321	PGBD1
Ectatosticta_davidi_00000449	PGBD2
Ectatosticta_davidi_00005156	PGBD2
Ectatosticta_davidi_00011992	PGBD2
Ectatosticta_davidi_00010106	PGBD3
Ectatosticta_davidi_00000872	PGBD3
Ectatosticta_davidi_00002520	PGBD3
Ectatosticta_davidi_00002659	PGBD3
Ectatosticta_davidi_00002862	PGBD3
Ectatosticta_davidi_00003912	PGBD3
Ectatosticta_davidi_00003924	PGBD3
Ectatosticta_davidi_00007521	PGBD3
Ectatosticta_davidi_00008387	PGBD3
Ectatosticta_davidi_00009062	PGBD3
Ectatosticta_davidi_00009505	PGBD3
Ectatosticta_davidi_00010563	PGBD3
Ectatosticta_davidi_00010729	PGBD3
Ectatosticta_davidi_00011985	PGBD3
Ectatosticta_davidi_00012296	PGBD3
Ectatosticta_davidi_00012718	PGBD3
Ectatosticta_davidi_00013299	PGBD3
Ectatosticta_davidi_00013507	PGBD3
Ectatosticta_davidi_00015104	PGBD3
Ectatosticta_davidi_00009536	PGBD4
Ectatosticta_davidi_00000024	PGBD4
Ectatosticta_davidi_00001772	PGBD4
Ectatosticta_davidi_00002245	PGBD4
Ectatosticta_davidi_00002553	PGBD4
Ectatosticta_davidi_00002641	PGBD4
Ectatosticta_davidi_00003044	PGBD4
Ectatosticta_davidi_00003719	PGBD4
Ectatosticta_davidi_00004846	PGBD4
Ectatosticta_davidi_00006316	PGBD4
Ectatosticta_davidi_00006338	PGBD4
Ectatosticta_davidi_00006433	PGBD4
Ectatosticta_davidi_00006785	PGBD4
Ectatosticta_davidi_00007000	PGBD4
Ectatosticta_davidi_00007571	PGBD4
Ectatosticta_davidi_00009132	PGBD4
Ectatosticta_davidi_00009266	PGBD4
Ectatosticta_davidi_00009470	PGBD4
Ectatosticta_davidi_00009478	PGBD4
Ectatosticta_davidi_00010406	PGBD4
Ectatosticta_davidi_00010724	PGBD4
Ectatosticta_davidi_00010765	PGBD4
Ectatosticta_davidi_00010767	PGBD4
Ectatosticta_davidi_00012022	PGBD4
Ectatosticta_davidi_00012883	PGBD4
Ectatosticta_davidi_00012948	PGBD4
Ectatosticta_davidi_00013644	PGBD4
Ectatosticta_davidi_00015437	PGBD4
Ectatosticta_davidi_00015628	PGBD4

Supplementary Table 7. The number of GMC genes in the different species.

Species	Number	Note	Cite
Ectatosticta davidi	44	spider	this work
Argiope bruennichi	27	spider	this work
Caerostris darwini	19	spider	this work
Caerostris extrusa	30	spider	this work
Nephila pilipes	34	spider	this work
Parasteatoda tepidariorum	37	spider	this work
Stegodyphus dumicolals	14	spider	this work
Stegodyphus mimosarum	13	spider	this work
Trichonephila antipodiana	16	spider	this work
Trichonephila clavipes	25	spider	this work
Drosophila melanogaster	15	insect	(Iida et al., 2007)
Bombyx mori	43	insect	(Sun et al., 2012)
Danaus plexippus	33	insect	(Sun et al., 2012)
Tribolium castaneum	23	insect	(Iida et al., 2007)
Apis mellifera	18	insect	(Iida et al., 2007)

Supplementary Table 8. Spidrion genes in *E. davidi*.

Gene ID	subfamily	Position
Ectatosticta_davidi_00004156	TuSp	Chr12: 118536334-118538331
Ectatosticta_davidi_00014541	Masp	Chr8: 65115938-65132430
Ectatosticta_davidi_00014568	AcSp	Chr8: 69276896 69299266
Ectatosticta_davidi_00014990	CrSp	Chr10: 23353978-23372837

Supplementary Table 9. Annotation of all 45 toxin genes in *E. davidi* genome.

Query_id	Name	Protein type
Ectatosticta_davidi_00013042-RA	Eda_ALL7_1	ALL7
Ectatosticta_davidi_00013043-RA	Eda_ALL7_2	ALL7
Ectatosticta_davidi_00013040-RA	Eda_ALL7_3	ALL7
Ectatosticta_davidi_00013044-RA	Eda_ALL7_4	ALL7
Ectatosticta_davidi_00013041-RA	Eda_ALL7_5	ALL7
Ectatosticta_davidi_00013045-RA	Eda_ALL7_6	ALL7
Ectatosticta_davidi_00014006-RA	Eda_ALL7_7	ALL7
Ectatosticta_davidi_00014199-RA	Eda_ALL7_8	ALL7
Ectatosticta_davidi_00014197-RA	Eda_ALL7_9	ALL7
Ectatosticta_davidi_00014198-RA	Eda_ALL7_10	ALL7
Ectatosticta_davidi_00003851-RA	Eda_ALL7_11	ALL7
Ectatosticta_davidi_00014196-RA	Eda_ALL7_12	ALL7
Ectatosticta_davidi_00014200-RA	Eda_ALL7_13	ALL7
Ectatosticta_davidi_00001840-RA	Eda_ALL7_14	ALL7
Ectatosticta_davidi_00014003-RA	Eda_ALL7_15	ALL7
Ectatosticta_davidi_00009885-RA	Eda_CRISPs_1	CRISPs
Ectatosticta_davidi_00009598-RA	Eda_CRISPs_2	CRISPs
Ectatosticta_davidi_00009599-RA	Eda_CRISPs_3	CRISPs
Ectatosticta_davidi_00015199-RA	Eda_CRISPs_4	CRISPs
Ectatosticta_davidi_00006893-RA	Eda_CRISPs_5	CRISPs
Ectatosticta_davidi_00008580-RA	Eda_CRISPs_6	CRISPs
Ectatosticta_davidi_00008579-RA	Eda_CRISPs_7	CRISPs
Ectatosticta_davidi_00014681-RA	Eda_CRISPs_8	CRISPs
Ectatosticta_davidi_00014682-RA	Eda_CRISPs_9	CRISPs
Ectatosticta_davidi_00007014-RA	Eda_CRISPs_10	CRISPs
Ectatosticta_davidi_00005759-RA	Eda_SMase-4_1	SMase-
Ectatosticta_davidi_00005760-RA	Eda_SMase-4_2	4SMase-
Ectatosticta_davidi_00007160-RA	Eda_SMase-4_3	4SMase-4
Ectatosticta_davidi_00010507-RA	Eda_AK_1	AK
Ectatosticta_davidi_00014216-RA	Eda_AK_2	AK
Ectatosticta_davidi_00015485-RA	Eda_AK_3	AK
Ectatosticta_davidi_00011193-RA	Eda_ACE_1	ACE
Ectatosticta_davidi_00011194-RA	Eda_ACE_2	ACE
Ectatosticta_davidi_00013758-RA	Eda_ACE_3	ACE
Ectatosticta_davidi_00005614-RA	Eda_PA2_1	PA2
Ectatosticta_davidi_00004512-RA	Eda_PA2_2	PA2
Ectatosticta_davidi_00009982-RA	Eda_PA2_3	PA2
Ectatosticta_davidi_00000365-RA	Eda_PA2_4	PA2
Ectatosticta_davidi_00015319-RA	Eda_PA2_5	PA2
Ectatosticta_davidi_00011781-RA	Eda_PA2_6	PA2
Ectatosticta_davidi_00015318-RA	Eda_PA2_7	PA2
Ectatosticta_davidi_00010233-RA	Eda_PA2_8	PA2
Ectatosticta_davidi_00000752-RA	Eda_PA2_9	PA2
Ectatosticta_davidi_00011775-RA	Eda_PA2_10	PA2
Ectatosticta_davidi_00000422-RA	Eda_HYAL_1	HYAL

Supplementary Table 10. Numerical statistics of spider toxin genes in different species.

Species	CRISPs	AK	ALL7	PA2	SMase D	ACE	HYAL	latrotoxin	lycotoxin	Total	Reference
Ectatosticta davidi	10	3	15	10	3	3	1	0	0	45	This work
Trichonephila antipodiana	14	2	14	13	4	7	0	0	1	55	This work
Hylyphantes graminicola	16	3	12	11	7	6	1	2	1	59	(Zhu et al., 2022)
Argiope bruennichi	16	3	14	11	5	7	0	0	7	63	(Sheffer et al., 2021)
Parasteatoda tepidariorum	9	2	6	9	5	6	1	45	1	84	(Schwager et al., 2017)
Stegodyphus dumicola	9	2	4	14	6	3	0	0	0	38	(Sanggaard et al., 2014)
Stegodyphus mimosarum	9	2	7	11	4	5	0	0	0	38	(Sanggaard et al., 2014)
Centruroides sculpturatus	17	1	9	15	5	4	2	0	0	53	(Zhu et al., 2022)
Hyalomma asiaticum	0	3	9	5	8	4	0	0	0	29	(Zhu et al., 2022)
Tetranychus urticae	0	1	1	5	4	1	0	0	0	12	（Hedges et al., 2006)
Tribolium castaneum	26	2	4	6	0	5	1	0	0	44	（Hedges et al., 2006)
Drosophila melanogaster	25	4	0	6	0	6	0	0	0	41	（Hedges et al., 2006)
Vespula pensylvanica	2	1	1	4	0	3	1	0	0	12	（Hedges et al., 2006)
