# Supplementary material for: Lampshade web spider Ectatosticta davidi chromosome-level genome assembly provides evidence for its phylogenetic position
Source: Commun Biol. 2023 Jul 18;6:748. doi: 10.1038/s42003-023-05129-x (PMC10354039; doi:10.1038/s42003-023-05129-x)
Supplement: Supplementary file 2 — Reporting Summary [file 42003_2023_5129_MOESM2_ESM.pdf]

Corresponding author(s): DAPR COMMSBIO-22-4334A

Last updated by author(s): Jun 25, 2023

# Reporting Summary

Nature Portfolio wishes to improve the reproducibility of the work that we publish. This form provides structure for consistency and transparency in reporting. For further information on Nature Portfolio policies, see our Editorial Policies and the Editorial Policy Checklist.

## Statistics

For all statistical analyses, confirm that the following items are present in the figure legend, table legend, main text, or Methods section.

| n/a | Confirmed | |
|---|---|---|
| ☐ | ☒ | The exact sample size (*n*) for each experimental group/condition, given as a discrete number and unit of measurement |
| ☐ | ☒ | A statement on whether measurements were taken from distinct samples or whether the same sample was measured repeatedly |
| ☐ | ☒ | The statistical test(s) used AND whether they are one- or two-sided<br>*Only common tests should be described solely by name; describe more complex techniques in the Methods section.* |
| ☐ | ☒ | A description of all covariates tested |
| ☐ | ☒ | A description of any assumptions or corrections, such as tests of normality and adjustment for multiple comparisons |
| ☐ | ☒ | A full description of the statistical parameters including central tendency (e.g. means) or other basic estimates (e.g. regression coefficient) AND variation (e.g. standard deviation) or associated estimates of uncertainty (e.g. confidence intervals) |
| ☐ | ☒ | For null hypothesis testing, the test statistic (e.g. *F*, *t*, *r*) with confidence intervals, effect sizes, degrees of freedom and *P* value noted<br>*Give P values as exact values whenever suitable.* |
| ☐ | ☒ | For Bayesian analysis, information on the choice of priors and Markov chain Monte Carlo settings |
| ☐ | ☒ | For hierarchical and complex designs, identification of the appropriate level for tests and full reporting of outcomes |
| ☐ | ☒ | Estimates of effect sizes (e.g. Cohen's *d*, Pearson's *r*), indicating how they were calculated |

*Our web collection on statistics for biologists contains articles on many of the points above.*

## Software and code

Policy information about availability of computer code

| | |
|---|---|
| Data collection | SRA Toolkit v3.0.1 was used to download the SRA data from NCBI. |
| Data analysis | BBTools suit V38.67, GenomeScope v1.0.0, Raven v1.6.1, Purge Hapolotigs v1.1.0, Nextpolish v1.3.1, Minimap2 v2.1.2, Juicer v1.6.2, blast+ v2.7.1, RepeatModeler v2.0.2, RepeatMasker v4.1.2, Maker pipline v3.01.03, HISAT2 v2.2.1, Striingtie v2.1.6, Augustus v3.4.1, GeneMarker-ES/ET/EP v4.68_lic, BRAKER v2.16, EggNOG-mapper v2.1.5, Diamond v2.0, InterProscan v5.48-83.0, Infernal v1.1.4, tRNAscan-SE v2.0, BUSCO V5.2.2, Orthofinder v2.5.4, Mafft v7.487. trimAL v1.4, FASconCAT-G V1.04, IQ-TREE v2.1.3, PAML package v4.9j, CAFE v4.2.1, and CAFE5, BITACORA pipline, MMseqs2 v11, Figtree v1.4.3, Evolview v3 webserver, MG2C, MCScanX, featureCounts v1.6.4 |

For manuscripts utilizing custom algorithms or software that are central to the research but not yet described in published literature, software must be made available to editors and reviewers. We strongly encourage code deposition in a community repository (e.g. GitHub). See the Nature Portfolio guidelines for submitting code & software for further information.

# Data

Policy information about availability of data

All manuscripts must include a data availability statement. This statement should provide the following information, where applicable:

- Accession codes, unique identifiers, or web links for publicly available datasets
- A description of any restrictions on data availability
- For clinical datasets or third party data, please ensure that the statement adheres to our policy

The sequencing data sets supporting the results of this article are available in NCBI (BioProject ID PRJNA853523), Illumina data with SRR19913594, PacBio data with SRR20336950, Hi-C data with SRR19905029, RNA data with SRR19913735.

## Research involving human participants, their data, or biological material

Policy information about studies with human participants or human data. See also policy information about sex, gender (identity/presentation), and sexual orientation and race, ethnicity and racism.

| | |
|---|---|
| Reporting on sex and gender | not involved |
| Reporting on race, ethnicity, or other socially relevant groupings | not involved |
| Population characteristics | not involved |
| Recruitment | not involved |
| Ethics oversight | not involved |

Note that full information on the approval of the study protocol must also be provided in the manuscript.

# Field-specific reporting

Please select the one below that is the best fit for your research. If you are not sure, read the appropriate sections before making your selection.

☐ Life sciences ☐ Behavioural & social sciences ☒ Ecological, evolutionary & environmental sciences

For a reference copy of the document with all sections, see nature.com/documents/nr-reporting-summary-flat.pdf

# Ecological, evolutionary & environmental sciences study design

All studies must disclose on these points even when the disclosure is negative.

| | |
|---|---|
| Study description | Chromosome-level genome assembly of the lampshade web spider Ectatosticta davidi providence for its phylogenetic position. |
| Research sample | Female specimens of E.davidi were collected and used for genome sequencing. |
| Sampling strategy | Female |
| Data collection | the sample was sent to the Berry Genomics (Beijing, China)Company for genome sequencing |
| Timing and spatial scale | not incolved |
| Data exclusions | not involved |
| Reproducibility | not involved |
| Randomization | not involved |
| Blinding | not involved |

Did the study involve field work? ☐ Yes ☒ No

# Reporting for specific materials, systems and methods

We require information from authors about some types of materials, experimental systems and methods used in many studies. Here, indicate whether each material, system or method listed is relevant to your study. If you are not sure if a list item applies to your research, read the appropriate section before selecting a response.

## Materials & experimental systems

| n/a | Involved in the study |
| --- | --- |
| ☒ | ☐ Antibodies |
| ☒ | ☐ Eukaryotic cell lines |
| ☒ | ☐ Palaeontology and archaeology |
| ☐ | ☒ Animals and other organisms |
| ☒ | ☐ Clinical data |
| ☒ | ☐ Dual use research of concern |
| ☒ | ☐ Plants |

## Methods

| n/a | Involved in the study |
| --- | --- |
| ☒ | ☐ ChIP-seq |
| ☒ | ☐ Flow cytometry |
| ☒ | ☐ MRI-based neuroimaging |

## Animals and other research organisms

Policy information about studies involving animals; ARRIVE guidelines recommended for reporting animal research, and Sex and Gender in Research

| | |
| --- | --- |
| Laboratory animals | not involved |
| Wild animals | The live spider was sent to the company for sequencing. The spiders were cleaned and ground in liquid nitrogen. |
| Reporting on sex | Though we used the female spider for genome sequencing, the genome analysis was not involved in sex. |
| Field-collected samples | The spider was collected from the Qinling Mountains, Chang'an District of Xi'an City, Shaanxi Province of China, in October 2021. |
| Ethics oversight | No ethical approval, because the study of the spider genome not involved in ethical. |

Note that full information on the approval of the study protocol must also be provided in the manuscript.
